# Investigating Host and Parasitic Plant Interaction by Tissue-Specific Gene Analyses on Tomato and *Cuscuta campestris* Interface at Three Haustorial Developmental Stages

**DOI:** 10.3389/fpls.2021.764843

**Published:** 2022-02-10

**Authors:** Min-Yao Jhu, Moran Farhi, Li Wang, Kristina Zumstein, Neelima R. Sinha

**Affiliations:** ^1^Department of Plant Biology, University of California, Davis, CA, United States; ^2^Crop Science Centre, Department of Plant Sciences, University of Cambridge, Cambridge, United Kingdom; ^3^The Better Meat Co., West Sacramento, CA, United States; ^4^College of Life Sciences, Nanjing Normal University, Nanjing, China

**Keywords:** parasitic plants, *Cuscuta campestris*, laser-capture microdissection, host-induced gene silencing, CRISPR, haustoria, Solanum lycopersicum

## Abstract

Parasitic weeds cause billions of dollars in agricultural losses each year worldwide. *Cuscuta campestris* (*C. campestris*), one of the most widespread and destructive parasitic plants in the United States, severely reduces yield in tomato plants. Reducing the spread of parasitic weeds requires understanding the interaction between parasites and hosts. Several studies have identified factors needed for parasitic plant germination and haustorium induction, and genes involved in host defense responses. However, knowledge of the mechanisms underlying the interactions between host and parasitic plants, specifically at the interface between the two organisms, is relatively limited. A detailed investigation of the crosstalk between the host and parasite at the tissue-specific level would enable development of effective parasite control strategies. To focus on the haustorial interface, we used laser-capture microdissection (LCM) with RNA-seq on early, intermediate and mature haustorial stages. In addition, the tomato host tissue that immediately surround the haustoria was collected to obtain tissue- resolution RNA-Seq profiles for *C. campestris* and tomato at the parasitism interface. After conducting RNA-Seq analysis and constructing gene coexpression networks (GCNs), we identified *CcHB7*, *CcPMEI*, and *CcERF1* as putative key regulators involved in *C. campestris* haustorium organogenesis, and three potential regulators, *SlPR1*, *SlCuRe1-like*, and *SlNLR*, in tomatoes that are involved in perceiving signals from the parasite. We used host-induced gene silencing (HIGS) transgenic tomatoes to knock-down the candidate genes in *C. campestris* and produced CRISPR transgenic tomatoes to knock out candidate genes in tomatoes. The interactions of *C. campestris* with these transgenic lines were tested and compared with that in wild-type tomatoes. The results of this study reveal the tissue-resolution gene regulatory mechanisms at the parasitic plant-host interface and provide the potential of developing a parasite-resistant system in tomatoes.

## Introduction

Parasitic angiosperms are among the worst agricultural pests, reducing the yields of agricultural crops each year by billions of dollars worldwide (Agrios, 2005; Yoder and Scholes, 2010). Parasitic plants directly attach to host plants using specialized organs known as haustoria to extract nutrients and water from their hosts. Most standard herbicides and control techniques have not been effective or are too costly in managing parasitic plant infestations because of this tight physiological link between host plants and parasites. A better understanding of the mechanisms of parasitic signaling and haustorium development will allow us to develop more robust biocontrol approaches to eliminate the agricultural damage caused by parasitic plants.

*Cuscuta* species (dodders) lack functional roots and leaves and coil their stems counterclockwise as they grow on their host (Furuhashi et al., 2011; Alakonya et al., 2012). About 75% of *Cuscuta* species are found in the Americas (Furuhashi et al., 2011; García et al., 2014), including *Cuscuta campestris* (*C. campestris*). Many crop species are susceptible to *C. campestris* attack, including domesticated tomatoes (*Solanum lycopersicum*), leading to 50–72% yield reductions (Yaakov et al., 2001). In California, over 12,000 hectares of land are affected by *Cuscuta* (Lanini and Kogan, 2005). Tomato is one of the most consumed fruit crops in the world, and the United States is one of the world’s leading producers of tomatoes (Kimura and Sinha, 2008). In the United States, more than $2 billion in annual farm cash receipts are from fresh and processed tomatoes. Therefore, a detailed investigation of the haustorial development process in the interactions between tomato and *Cuscuta* is essential to developing effective strategies to prevent agricultural losses that are caused by *Cuscuta* species.

However, the signals involved in haustorium development at specific developmental stages and the tissue-specific communication between host and parasite during the haustorium penetration process remain largely unknown. This is especially true for stem parasitic plant systems. Several studies have indicated that haustoria can transport not only water and nutrients, but also mRNA, miRNA, and small peptides (Kim et al., 2014; Johnson et al., 2019; Shen et al., 2020). These bidirectional communications create a tight physiological connection between host and parasite. During the haustorium development process, parasitic plants change their host morphologically and physiologically by secreting hormones or effectors to help them establish haustorial connections (Shimizu and Aoki, 2019; Su et al., 2020). On the other hand, host plants deploy various defense strategies to counteract this infestation and prevent vascular connections (Fishman and Shirasu, 2021). Understanding what parasitism-related genes have been explicitly activated at the interface between host and parasite could help develop a more efficient parasite-resistant system in crop plants.

Therefore, in this study, we used laser-capture microdissection (LCM) coupled with RNA-seq to zoom in on the interface between the host and parasite and to investigate the tissue-specific gene expression changes. We identified *CcHB7*, *CcPMEI*, and *CcERF1* as key regulators involved in haustorium organogenesis, and the functions of these candidate genes were validated by HIGS transgenic plants. We also identified three potential key regulators, *SlPR1*, *SlCuRe1-like*, and *SlNLR*, in tomatoes that may be involved in perceiving signals from the parasites, and two of them were further characterized with CRISPR knockout mutants.

## Results

### Transcriptomes at the Host-Parasite Interface Using Laser-Capture Microdissection

**FIGURE 1:**
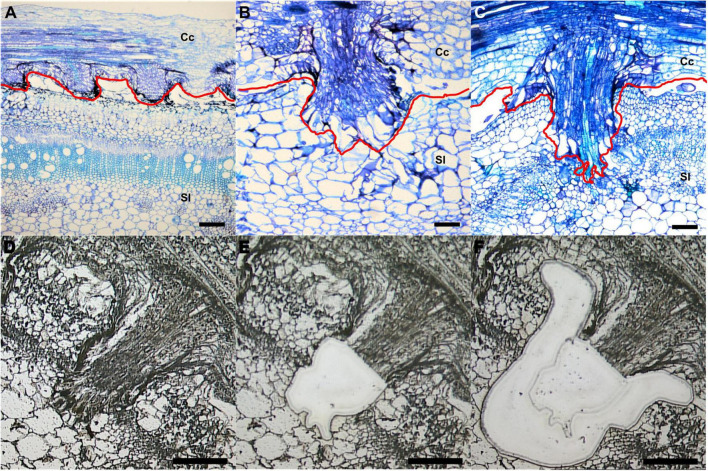
Laser-capture microdissection (LCM) of *C. campestris* haustoria penetrating tomato stems at three developmental stages. **(A–C)** Toluidine blue O stained paraffin sections of tomato stem with *C. campestris* early **(A)**, intermediate **(B)**, and mature stage **(C)** haustoria. Red line indicates the interface between *C. campestris* and host tomato. Cc indicates *C. campestris*; Sl indicates *S. lycopersicum*. **(D–F)** *C. campestris* haustorial tissues and host tissues were collected using LCM. A paraffin section of an intermediate stage haustorium before collection **(D)**, after haustorial tissue collection **(E)**, and after host tissue collection **(F)**. **(A)** and **(C)**, scale bars = 250 μm. **(B)**, **(D)**, **(E)**, and **(F)**, scale bars = 100 μm. Part of this figure **(A–C)** is modified from one in a previously published paper Jhu et al., 2021 with new information added.

To investigate specific gene expression changes in penetrating haustoria, we used LCM with RNA-seq to analyze haustorial tissues from three different developmental stages. We defined three time points: early - the haustorium has just contacted the host, intermediate - the haustorium has developed searching hyphae, which are elongated tip-growing cells on haustoria, but has not formed vascular connections, and mature - a haustorium with continuous vascular tissue between host and parasite (Figures 1A-C).

We collected both parasite haustorial tissues and host tissues at the interface at these three-time points from *C. campestris* attached on *S. lycopersicum* cv. Heinz 1706 (H1706). To identify genes involved in *C. campestris* haustorial development during the penetration process, the protruding regions of haustoria were specifically collected from paraffin sections using LCM (Figures 1D,E). To capture the earliest host responses or defense mechanisms to combat *C. campestris* parasitism, we choose the same three developmental time points, early, intermediate, and mature stages of parasitism, to specifically collect the few layers of tomato cells that surround the penetrating *C. campestris* haustoria (Figure 1F). These host cells are most likely to exhibit the initial host defense response upon attacks by the parasite. These collected tomato and *C. campestris* tissues were processed for RNA extraction and library preparation for RNA-Seq and subsequent transcriptome analysis.

### RNA-Seq Analyses and Gene Coexpression Networks Across Three Developmental Stages of *Cuscuta campestris* Haustoria

**FIGURE 2:**
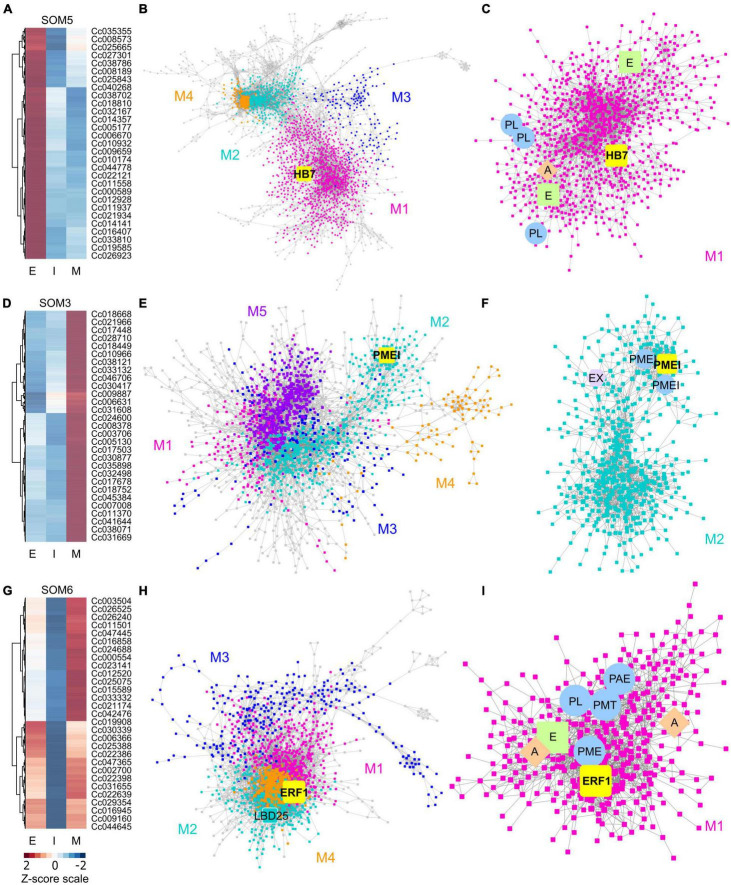
Heatmaps and gene-coexpression networks (GCNs) of *C. campestris* haustorial tissues across three developmental stages. **(A)** A heatmap of gene expression profiles in z-scores for SOM5, which includes genes that are highly expressed in the early stage. **(B)** A GCN of genes in SOM5. This SOM5 GCN is composed of four major modules. Magenta indicates genes in Module 1, which has enriched biological process GO term including “response to abiotic and biotic stimulus, stress, hormone, far-red light.” The transcription factor HB7 is enlarged and labeled in yellow. **(C)** GCN of genes that are classified in SOM5 Module 1. PL, pectin lyase-like superfamily protein. A, auxin response factor 1. E, ethylene signaling-related genes. **(D)** A heatmap of gene expression profiles in z-scores for SOM3, which includes genes that are highly expressed in the mature stage. **(E)** A GCN of genes that are in SOM3. The SOM3 GCN is composed of five major modules. Cyan indicates genes in Module 2, which has enriched biological process GO term including “root radial pattern formation.” Selected pectin methyl-esterase inhibitor (PMEI) is enlarged and labeled in yellow. **(F)** GCN of genes that are classified in SOM3 Module 2. PMEI, pectin methyl-esterase inhibitor. EX, expansin. **(G)** A heatmap of gene expression profiles in z-scores for SOM6, which includes genes that are relatively highly expressed in the early and mature stage. **(H)** A GCN of genes in SOM6. This SOM6 GCN is composed of four major modules. Magenta indicates genes in Module 1, which has enriched biological process GO term including “response to stimulus, hormone, organic substance.” Ethylene responsive element binding factor 1 (ERF1) is enlarged and labeled in yellow. The transcription factor LBD25 is enlarged and labeled in cyan as other genes in Module 2. **(I)** GCN of genes that are classified in SOM6 Module 1. PL, pectin lyase-like superfamily protein. A, auxin transporter or auxin-responsive protein; E, ethylene signaling-related genes; PL, pectin lyase-like superfamily protein; PME, pectin methylesterase; PMT, pectin methyltransferase-like; PAE, pectin acetylesterase family protein. The complete gene lists for all SOM units with SOM distances and PCA principal component values are included in Supplementary Table 1. The selected SOM gene lists were used for constructing the GCN based on the expression profiles in *C. campestris* LCM data with the following normal quantile cutoffs. The SOM5 GCN cutoff = 0.97. The SOM3 GCN cutoff = 0.98. The SOM6 GCN cutoff = 0.95.

We analyzed the transcriptome of LCM *C. campestris* haustorial tissues by mapping reads to the genome of *C. campestris* (Vogel et al., 2018). With multidimensional scaling analysis, the gene expression patterns among different samples showed that the early and intermediate stages are distinct from the mature stage (Supplementary Figure 1). We conducted a principal component analysis (PCA) and coupled it with clustering analysis using self-organizing maps (SOM) to visualize the expression profile of each gene (Supplementary Table 1 and Supplementary Figure 2). To identify potential key regulators that are involved in *C. campestris* haustorium development at different stages, we conducted further gene coexpression analysis on specific SOM clusters (Figure 2 and Supplementary Figure 3).

First, we hypothesized that the genes highly up-regulated at the early stage of parasitism are most likely to be involved in the haustorium initiation and attachment process. Therefore, we focused on genes in SOM5, which is a cluster enriched with genes that are highly expressed in the early haustorial stage. Among these SOM5 genes, we constructed a gene coexpression network (GCN) to generate an overview of the potential molecular regulatory machinery and to identify central hub genes, which are genes with high degree of centrality in the coexpression network, that could be the regulators of the initiation and attachment mechanisms. Visualizing the network using Cytoscape (Cline et al., 2007), the SOM5 GCN is composed of four major modules (Figures 2A–C and Supplementary Table 2) based on the fast greedy modularity optimization algorithm (Clauset et al., 2004). Using our previously published combined annotation profile, we conducted GO enrichment analysis using the matched TAIR ID for each *C. campestris* gene in the network to identify the major GO terms (FDR-values < 0.05) for the target modules. We find the SOM5 module 1 enriched in biological process GO terms including “response to abiotic and biotic stimulus, response to stress, response to hormone, and response to far-red light” (Supplementary Table 5). This result indicates the genes contained in module 1 are likely involved in the haustorium initiation process, which is responding to physical contact with the host and high far-red light environments, which are both important signals for haustorium induction in *Cuscuta* species.

To identify the key regulators in the haustorium initiation, we focused on genes in module 1 and calculated the degree centrality and betweenness centrality scores of each gene within this group because these gene are the potential master regulators. Many central hub genes in module 1 are protein kinases or enzymes involved in cell signaling. We focused on genes that are annotated as transcription factors to identify the potential master upstream key regulators of this developmental stage. Among these central hub transcription factors, a homeobox-leucine zipper protein (Cc014209) that is similar to transcription factor homeobox 7 (HB7) in *Arabidopsis* was identified (Figure 2B). Based on previous reports, *AtHB7* is a negative regulator of ABA response (Pehlivan, 2019) and modulates abscisic acid (ABA) signaling by controlling the activity of protein phosphatases type 2C (PP2C) and ABA receptors (Valdés et al., 2012). Intriguingly, a recent study showed that ABA levels regulate haustoria formation in the root parasitic plant *Phtheirospermum japonicum* (Kokla et al., 2021). In *P. japonicum*, lowering ABA biosynthesis enabled haustoria to form in the presence of nitrates. Based on these pieces of evidence, we focus on *CcHB7* for further functional analysis.

Second, the genes highly up-regulated at the mature stage are most likely to be involved in establishment of vascular connection between host and parasite. Therefore, we also focused on genes in SOM3, which is a cluster enriched with genes that are highly expressed in the mature haustorial stage (Figure 2D). Using these SOM3 genes, we constructed a GCN to generate an overview of the potential molecular regulatory machinery. This SOM3 GCN is composed of five major modules based on the GCN community structure (Figure 2E and Supplementary Table 3). Using GO enrichment analysis, we noticed that the SOM3 module 2 has enriched biological process GO terms including “root radial pattern formation” (Supplementary Table 5). This result matches our recent discovery that *C. campestris* also utilizes the root developmental program during haustorium organogenesis (Jhu et al., 2021). Therefore, we focused on SOM3 module 2 for further analysis. Based on the degree centrality and betweenness centrality scores, we noticed that many central hub genes in SOM3 module 2 are involved in cell wall modification, including expansins and several pectin methyl-esterase inhibitors (PMEIs) (Figure 2F). *CcPMEI* (Cc038093) is one of the central hub genes and has strong co-expression connection with other PMEIs. Thus, we focused on this *CcPMEI* for further functional analysis.

Last but not least, the previously identified key regulator in *C. campestris* haustorium development, transcription factor *LATERAL ORGAN BOUNDARIES DOMAIN 25* (*CcLBD25*) (Jhu et al., 2021) is classified in SOM6, which is enriched with genes that are highly expressed in the early and mature haustorial stage (Figure 2G). The SOM6 GCN is composed of four major modules based on the GCN community structure (Figure 2H and Supplementary Table 4). *CcLBD25* is located in module 2, which does not have any significantly enriched biological process GO terms (Supplementary Table 5). On the other hand, we noticed module 1 has enriched biological process GO term including “response to stimulus, response to hormone, response to organic substance.” Based on previous studies, auxin (Tomilov et al., 2005; Ishida et al., 2016) and ethylene (Cui et al., 2020) signaling play essential roles in parasitic plant haustorium development. Appropriate tactile stimuli, which come from the pressure coiling on the host, are also crucial for haustorium induction (Tada et al., 1996). Therefore, we zoomed in on SOM6 module 1 for further GCN analysis.

Many genes in SOM6 module 1 are enzymes that catalyze the degradation or modification of pectin, including pectin lyase (PL), pectin methyl esterase (PME), pectin methyltransferase (PMT), and pectin acetyl esterase (PAE) (Figure 2I). These findings coincide with several previous studies that cell wall modification, especially pectin structural dynamic and integrity, plays an important role in haustorium development (Vaughn, 2002; Johnsen et al., 2015; Hozumi et al., 2017). Besides cell wall remodeling, auxin and ethylene signaling also seem to play a role in the early and mature developmental stages. An auxin efflux carrier (Cc034373) and an auxin-responsive protein (Cc038909) were also located in SOM6 module 1 (Figure 2I). Furthermore, among the central hub genes in SOM6 module 1, the top central hub transcription factor is an ethylene responsive element binding factor 1 (*ERF1*, Cc002541), which is in the ERF/AP2 domain-containing transcription factor family. Intriguingly, a recent study showed that the root parasitic plant *Phtheirospermum japonicum* uses ethylene as a signal for host recognition and to tweak the haustorium development and penetration process (Cui et al., 2020). Our gene coexpression analysis suggests that this ethylene-mediated haustorial development regulatory pathway might be shared by both root and stem parasitic plants. Therefore, we focused on this *CcERF1* for further functional analysis.

### Functional Characterization of Candidate *Cuscuta campestris* Genes by Host-Induced Gene Silencing

Since an efficient transformation system for *C. campestris* is currently not available, to further validate the function of these candidate *C. campestris* genes, *CcHB7*, *CcPMEI*, and *CcERF1*, we used host-induced gene silencing (HIGS) to knock-down the candidate genes in *C. campestris.* Based on previous studies, cross-species transport of mRNAs, miRNAs and siRNAs between *C. campestris* and their hosts through haustoria vascular connections is common (Kim et al., 2014; Johnson et al., 2019). These transported siRNAs can successfully down-regulate target gene transcription in *C. campestris*, *via* HIGS. Therefore, we generated transgenic tomatoes with hairpin RNAi constructs that target and down-regulate the candidate *C. campestris* genes, *CcHB7*, *CcPMEI*, and *CcERF1*, after the first successful attachment. If these genes are important in haustorium development, down-regulating these genes should influence haustorium penetration and parasitism. We collected *C. campestris* haustorium and prehaustorium tissues next to the attachment sites and validated by qPCR that *CcHB7*, *CcPMEI*, and *CcERF1* are successfully knocked down in *C. campestris* tissues grown on HIGS transgenic tomatoes (Supplementary Figures 4A,C,D).

**FIGURE 3:**
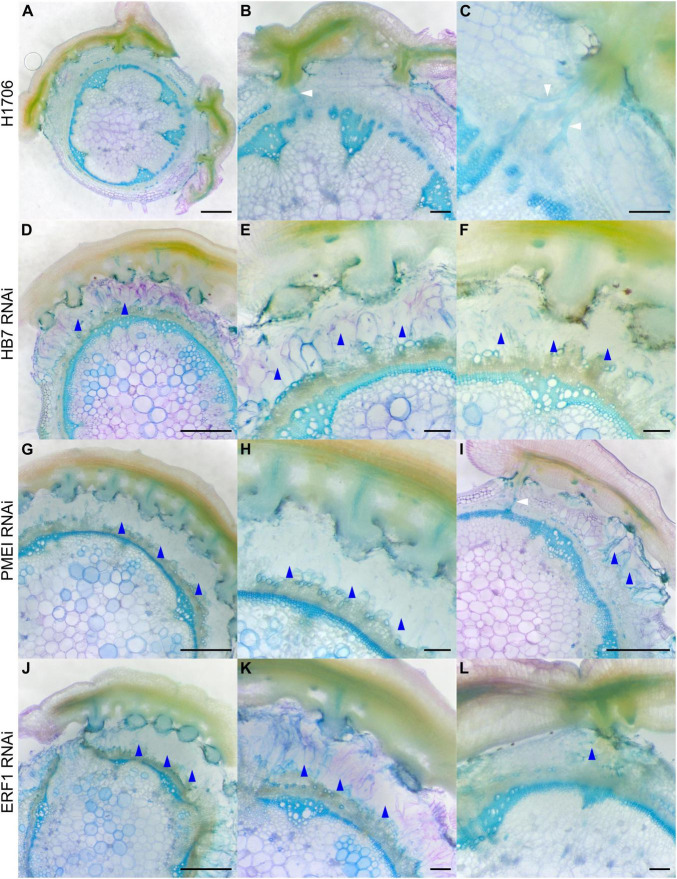
Haustorium phenotypes of *C. campestris* growing on Heinz tomato wild-types and HIGS RNAi transgenic plants. *C. campestris* haustoria that were growing on wild-type H1706 tomato hosts **(A–C)**, on HB7 RNAi transgenic tomato plants **(D–F)**, on PMEI RNAi transgenic tomato plants **(G–I)**, on ERF1 RNAi transgenic tomato plants **(J–L)**. **(A,D,G,I,J)** Scale bars = 1 mm. **(B,C,E,F,H,K,L)** Scale bars = 200 μm. **(A–L)** 100 μm thick vibratome sections of fresh haustorium stained with Toluidine Blue O. White arrowhead indicates normal haustorial vascular connections. Blue arrowhead indicates the phenotype of overly degraded host cortex cell walls.

To determine if these genes impact haustorium structure or the parasitism process, we grew *C. campestris* strands on the HIGS transgenic tomato. We collected tomato stem sections with *C. campestris* strands successfully attached on them and used vibratome sectioning to prepare 100 μm-thick fresh haustorium sections and subsequently stained them with Toluidine Blue O (O’Brien et al., 1964). We observed searching hyphae that entered the host cortex successfully and converted into xylic hyphae, which create the xylem bridge between host and parasite, or phloic hyphae, which mimic sieve elements and establish phloem-to-phloem connections, as they linked to the host xylem and phloem in sections of the haustoria growing on wild-type H1706 tomato plants (Figures 3A–C). However, we observed that many haustoria growing on *CcHB7* RNAi (Figures 3D–F), *CcPMEI* RNAi (Figures 3G–I), and *CcERF1* RNAi (Figures 3J–L) transgenic tomatoes seems to stop their penetration process at the cortex region. Furthermore, they also all shared a common phenotype that the host cortex cells that are surrounding the haustoria seem to enlarge and have a very loose cell wall structure, appearing degraded (Figures 3D–L). These *C. campestris* haustoria were not able to form vascular connections with their hosts and easily detached from their host stems.

This structural phenotype also corresponds well with our GCN results, especially the GCN for SOM3 and SOM6 (Figure 2). Several previous studies indicate that the interaction between pectin methyl esterase (PME) and pectin methyl esterase inhibitor (PMEI) is a determinant factor in pectin degradation, cell wall loosening, strengthening, and organogenesis. Pectin acetyl esterases (PAEs) are involved in the enzymatic deacetylation of pectin and are used by plant pathogens to infect their hosts (Kong et al., 2019). However, the balance between different pectin enzyme functions might be precisely regulated by many key regulators. The down-regulation of these key regulators might disrupt the dynamic balance between enzymes and cause an out-of-control cell wall degradation, which lead to haustorium detachment from its host (Figures 3D–L).

**FIGURE 4:**
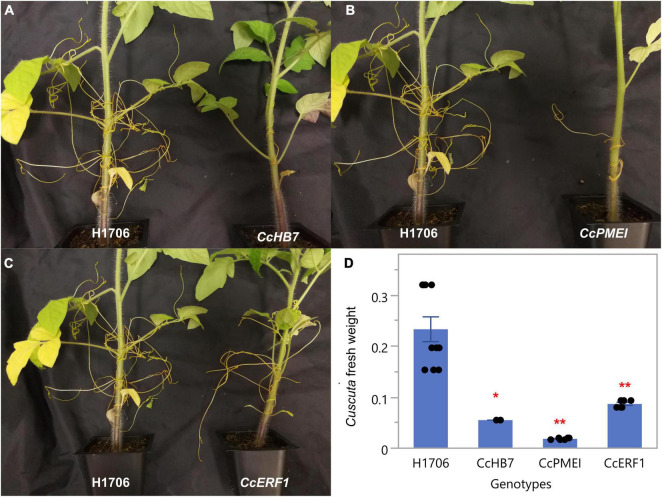
Phenotypes of *C. campestris* growing on Host-Induced Gene Silencing (HIGS) RNAi transgenic plants. **(A)** *C. campestris* growing on wild-type H1706 tomatoes and T1 *CcHB7* RNAi transgenic plants. **(B)** *C. campestris* growing on wild-type H1706 tomatoes and T1 *CcPMEI* RNAi transgenic plants. **(C)** *C. campestris* growing on wild-type H1706 tomatoes and T1 *CcERF1* RNAi transgenic plants. **(D)** Averaged biomass of *C. campestris* growing on wild-type H1706 tomatoes and T1 *CcHB7, CcPMEI*, and *CcERF1*RNAi transgenic plants. Fresh net weights of *C. campestris* were measured in grams (g). Each data point represents one tomato plant (biological replicate). Data presented are assessed using Dunnett’s test with wild-type H1706 as control. “*” *p*-value < 0.0005. “**” *p*-value < 0.0001.

Notably, since these plants are in a HIGS system, the first successful haustorial connection is necessary for the small interfering RNAs to transfer from the host to the parasite. Therefore, we often observed a successfully connected haustorium followed by several abnormal haustorium attachments, including the phenotype of overly degraded host cortex cell walls (Figure 3I). The overall plant phenotypes of *C. campestris* growing on the HIGS transgenic plants also showed very few haustorial connections and the inability to continue to form more attachments with the hosts compared to those growing on wild-type H1706 tomato plants (Figures 4A–C). In addition, our preliminary biomass measurements also showed that the *C. campestris* plants growing on RNAi transgenic plants have reduced biomass compared with those growing on wild-type tomato plants (Figure 4D). All of these results indicate that the down-regulation of these candidate gene expression levels by HIGS interfered with haustorium development and hampered *C. campestris* parasitism.

### RNA-Seq Analyses and Gene Coexpression Networks Across Three Developmental Stages of Host Tissues Surrounding *Cuscuta campestris* Haustoria

**FIGURE 5:**
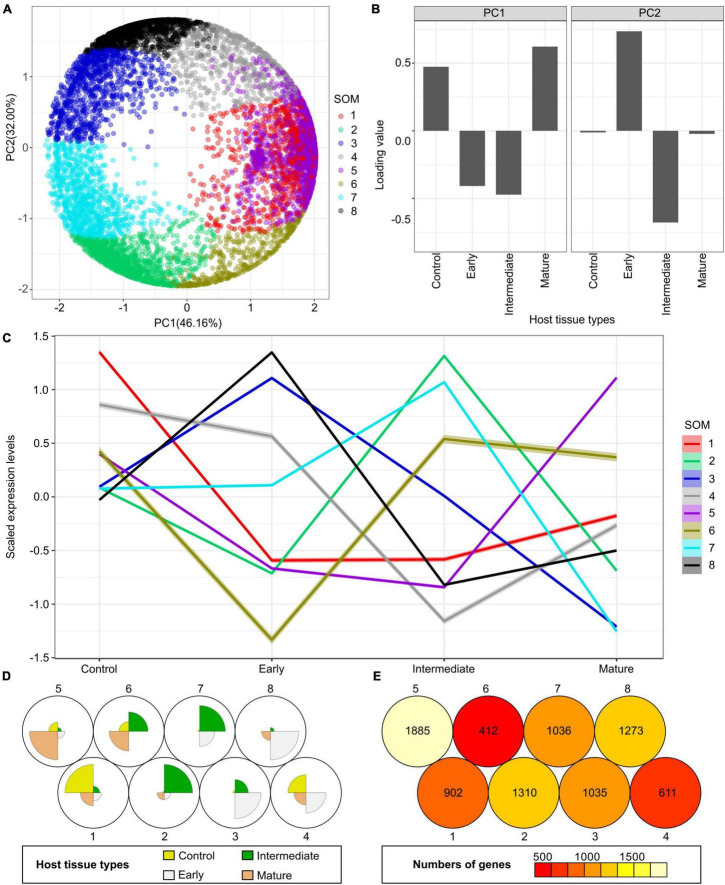
Principal component analysis (PCA) and self-organizing maps (SOM) clustering of LCM RNA-Seq data from the host tomato tissues surrounding *C. campestris* haustoria. **(A)** PCA plot of the first and second principal components (PC1 and PC2) and colored indicate their corresponding SOM groups. Each dot represents a gene. **(B)** Loading values of PC1 and PC2. “Control” means the tomato stem cortex tissue samples that are not next to *C. campestris* haustoria, which serve as negative controls in this experiment. PC1 separates the genes specifically expressed in host tissues sounding the early and intermediate-stage haustoria from those specifically expressed in other stages. PC2 separates the genes specifically expressed in host tissues surrounding the intermediate-stage haustoria from those expressed explicitly at the mature stage. **(C)** A plot of each SOM group’s scaled expression levels at four types of host tomato tissue surrounding *C. campestris* haustoria at different developmental stages. The color of each line represents the SOM group it belongs to. The shaded area around the lines indicates the 95 percent confidence interval. **(D)** A code plot of SOM clustering showing which developmental stage predominantly expresses genes of each SOM group based on sector size. Each sector represents the host tissues surrounding *C. campestris* haustoria at a specific developmental stage and is colored according to the tissue types it represents in figure legends. The number 1-8 next to each circle represents the corresponding SOM group. **(E)** A count plot of SOM clustering represents how many genes showed differential expression in each SOM group. The numbers of genes are labeled inside each circle representing SOM. **(D,E)** The number 1–8 next to each circle represents the corresponding SOM group.

**FIGURE 6:**
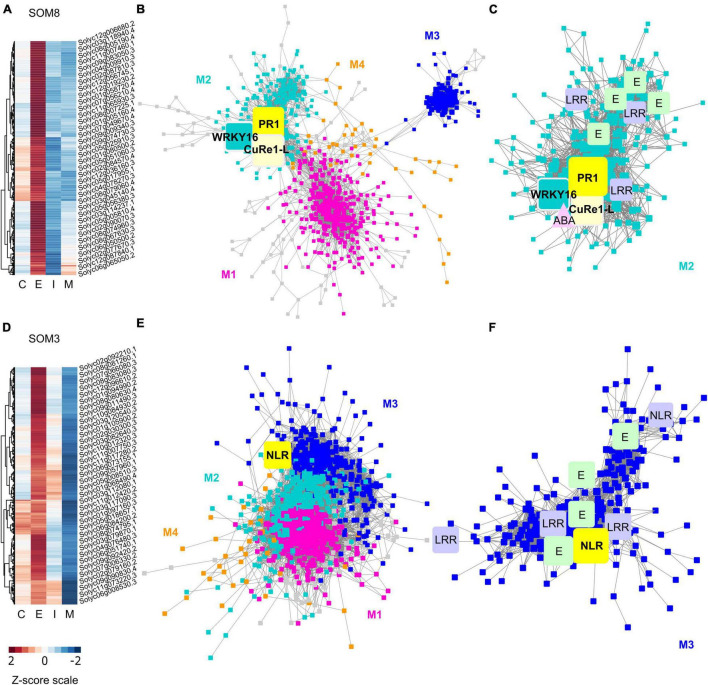
Heatmaps and gene-coexpression networks (GCNs) of LCM RNA-Seq data from the host tomato tissues surrounding *C. campestris* haustoria. **(A)** A heatmap of gene expression profiles in *z*-scores for SOM8, which includes genes specifically highly expressed in the early stage. **(B)** A GCN of genes in SOM8. This SOM8 GCN is composed of four major modules. Cyan indicates genes in Module 2. The transcription factor WRKY16 is enlarged and labeled in cyan as other genes in Module 2. PR1 is enlarged and labeled in yellow. CuRe1-like receptor (CuRe1-L) is enlarged and labeled in light yellow. **(C)** GCN of genes that are classified in SOM8 Module 2. CuRe1-L, CuRe1-like receptor. LRR, Leucine-rich repeat receptor-like protein kinase. ABA, Abscisic acid stress ripening 5. E, ethylene-responsive transcription factor. **(D)** A heatmap of gene expression profiles in z-scores for SOM3, including genes that have high expression levels in the early stage and moderate expression levels in the intermediate stage. **(E)** A GCN of genes in SOM3. This SOM3 GCN is composed of four major modules. Blue indicates genes in Module 3. NBS is enlarged and labeled in yellow. **(F)** GCN of genes that are classified in SOM3 Module 3. LRR, Leucine-rich repeat receptor-like protein kinase. E, ethylene-responsive transcription factor. NLR, NBS-LRR protein. The complete gene lists for all SOM units with SOM distances and PCA principal component values are included in Supplementary Table 6. The selected SOM gene lists were used for constructing the GCN based on the expression profiles in tomato LCM data with the following normal quantile cutoffs. The SOM8 GCN cutoff = 0.90. The SOM3 GCN cutoff = 0.80.

On the other side of this host-parasite interface is the tomato host. We analyzed the LCM RNA-Seq data from the host tomato tissues surrounding *C. campestris* haustoria by mapping reads to the tomato genome ITAG4.0 (Sato et al., 2012). Using multidimensional scaling analysis, the gene expression patterns in control host cell types (the regular tomato cortex cells that are not next to haustorium) are distinct from the cortex tissues surrounding *C. campestris* haustoria (Supplementary Figure 5). We also conducted a PCA coupled with SOM clustering analysis to visualize the expression profile of each gene (Figure 5 and Supplementary Table 6). To identify potential key regulators of the interaction between host and parasite at different haustorium penetration stages, we conducted further gene coexpression analysis on specific SOM clusters (Figure 6 and Supplementary Figure 6).

First, the host genes that are highly up-regulated at the early stage are most likely to be involved in perceiving parasite signals, triggering pattern-triggered immunity (PTI) and effector-triggered immunity (ETI) to help the host repel *C. campestris* attacks. Based on previous reports, the changes in the levels of salicylic acid (SA) and jasmonic acid (JA) in hosts are most obvious in the early stage [4 days post attachment (DPA)] (Runyon et al., 2010). So, we hypothesized that the most pronounced gene expression changes of key regulators would be at the initial stage of infestation. Therefore, we focused on genes in SOM8, which is a cluster enriched with host genes that are highly expressed in the early haustorial stage (Figure 6A). Among the genes in SOM8, we noticed inclusion of *SlWRKY16* (Solyc07g056280), a negative regulator of the lignin-based resistance response (Jhu et al., 2020). The SOM8 GCN is composed of four major modules based on the GCN community structure (Figure 6B and Supplementary Table 7). *SlWRKY16* is located in module 2. Based on our GO enrichment analysis, there are some genes that matched the GO term “regulation of defense response, and defense response to fungus,” but none of the GO terms were statistically significantly enriched in this module (Supplementary Table 9). However, one of the previously identified CuRe1 homologs (*CuRe1-like*, Solyc08g016210) (Fürst et al., 2016), which is a leucine-rich repeat (LRR) receptor-like serine/threonine-protein kinase, is also located in module 2 (Figure 6B). Therefore, we zoomed in on the SOM8 module 2 for further GCN analysis.

Many other genes in SOM8 module 2 are involved in ethylene signaling (Figure 6C). Ethylene is known to play a vital role in activating plant defenses against various biotic stresses, including microbial pathogens and herbivores (Broekgaarden et al., 2015). Previous studies also used the emission of ethylene in *N. benthamiana* and *S. lycopersicum* as an indicator that the defense response was successfully triggered upon *Cuscuta reflexa* infestation *(Hegenauer et al., 2016, 2020).* In addition to the CuRe1-like homolog, several LRR receptor-like protein kinases are also identified in SOM8 module 2 (Figure 6C). This result matches our hypothesis that the host genes that are highly up-regulated at the early stage are most likely to be involved in perceiving parasite signals. We suspect that *CuRe1-like* might play a role in sensing unknown *Cuscuta* signals, so we generated CRISPR-Cas9 edited *Cure1-like* mutant plants for further analysis.

Among the central hub genes in SOM8 module 2, *Pathogenesis-Related protein 1* (*PR1*, Solyc01g106600) was one of the top central hubs (*PR1* degree centrality = 64; median degree centrality in SOM8 = 13) (Figure 6C). PR1 proteins are known to be highly produced upon plant pathogen infection and have often been used as a marker for SA-mediated disease resistance (Breen et al., 2017). However, the role of PR1 in host plant responses upon parasitic plant attack is currently unknown. Therefore, we also focused on PR1 for further functional analysis using CRISPR-Cas9 gene editing.

Other than the host genes that are specifically only highly expressed at the early stage, another group of host genes are up-regulated at the early stage and gradually decrease their expression throughout parasitism. Genes with this expression pattern are found in SOM3 (Figure 6D). We suspected that these genes might also be involved in the parasite signal perceiving process. Hence, we focus on SOM3 for further GCN analysis. The SOM3 GCN is composed of four major modules based on the GCN community structure (Figure 6E and Supplementary Table 8). Based on our GO enrichment analysis, there are some genes in module 3 that matched the GO term “response to hormone,” but none of the GO terms were statistically significantly enriched in this module (Supplementary Table 9). However, many genes in SOM3 module 3 are LRR receptor-like kinases or nucleotide-binding site–leucine-rich repeat (NBS-LRR, or NLR) proteins. Therefore, we focused on SOM3 module 3 for further analysis.

Among the genes in SOM3 module 3, four genes involved in ethylene signaling were identified, including an ethylene-responsive transcription factor, an ethylene-responsive proteinase inhibitor and an ethylene-inducing xylanase receptor. This result provides further support for the hypothesis that ethylene might also play an important role in plant resistance responses against parasitic plants. In addition to the ethylene signaling pathway, potential transcription factors or receptors are also enriched in SOM3 module 3. Three LRR proteins and two NLRs are identified in module 3. NLRs are common disease resistance genes (R genes) and are known to be involved in biotic stress detection, including various plant pathogens and herbivores (McHale et al., 2006; Van Ghelder et al., 2019). Therefore, we suspected that these NLRs are potentially involved in the process of detecting parasitic plants signals and choose the NLR with the highest degree centrality (Solyc07g056200 degree centrality = 7; median degree centrality of NLRs in SOM3 module 3 = 6.5) in SOM3 module 3 as our candidate genes for further functional analysis.

### Functional Characterization of Tomato Host Genes by CRISPR-Cas9 Gene Editing

Since CRISPR knockout techniques (Pan et al., 2016) and tomato transformation systems are readily available, we designed and cloned synthetic guide RNAs (sgRNA) targeting our candidate genes, *SlPR1*, *SlCuRe1-like*, *SlNLR*, and then produced transgenic tomato plants in the M82 background with CRISPR/Cas9 targeted candidate gene knockout mutations. We selected the T0 transgenic plants that had biallelic insertion or deletion or substitution mutations for further T1 plants analysis (Supplementary Figure 6). After obtaining these CRISPR transgenic tomato lines, we tested the interactions of *C. campestris* with these engineered tomato lines and compared their *C. campestris*-host interactions with those seen in wild-type M82 tomatoes by phenotyping the haustorium attachment sites.

Interestingly, the *SlCuRe1-like* CRISPR T0 transgenic tomato lines were very vulnerable to pathogens and insect herbivores, did not grow well in our greenhouse conditions and only produced very few seeds. This indicates that *SlCuRe1-like* might play a role in the plant defense responses to other pathogens and herbivores in addition to any possible role in plant parasitism. As a result, due to low seed set we were unable to phenotype these CRISPR tomato lines, so *SlCuRe1-like* phenotyping is excluded from our current analysis.

**FIGURE 7:**
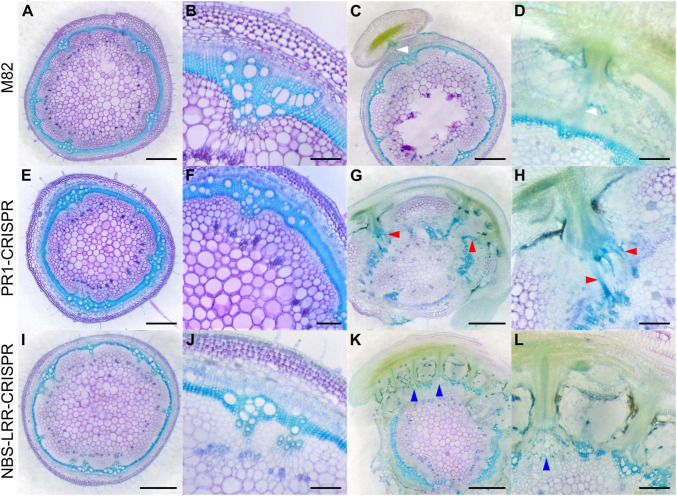
Haustorium phenotypes of *C. campestris* growing on M82 tomato wild-types and candidate genes-CRISPR transgenic plants. *C. campestris* haustoria that were growing on wild-type M82 tomato hosts **(A–D)**, on PR1-CRISPR T1 transgenic tomato plants **(E–H)**, on NBS-LRR-CRISPR T1 transgenic tomato plants **(I–L)**. **(G–H)** Vibratome sections of *C. campestris* growing on PR1-CRISPR T1 plant #6. **(K,L)** Vibratome sections of *C. campestris* growing on NBS-LRR-CRISPR T1 plant #4. **(A,C,E,G,I,K)** Scale bars = 1 mm. **(B,D,F,H,J,L)** Scale bars = 200 μm. **(A–L)** 100 μm thick vibratome sections of fresh haustorium stained with Toluidine Blue O. White arrowhead indicates normal haustorial vascular connections. Red arrowhead indicates the hypertrophy symptom with enlarged xylem bridges. Blue arrowhead indicates the phenotype of disrupted host stem vascular tissue arrangement.

When compared with wild-type M82 tomato plants (Figures 7A,B), *SlPR1* CRISPR T1 tomato plants (Figures 7E,F) and *SlNLR* CRISPR T1 tomato plants (Figures 7I,J) did not have an obvious difference in their overall plant or tissue phenotypes without *C. campestris* infestation, based on the fresh vibratome sections. However, the differences were apparent when these CRISPR tomato plants were infested by *C. campestris.* On wild-type M82 tomato plants, we observed that searching hyphae entered the host cortex and linked to the host xylem and phloem, but *C. campestris* did not change the overall host stem structure much, other than penetrating and forming vascular connections (Figures 7C,D).

In contrast, *SlPR1* CRISPR tomato plants seem to be more susceptible to *C. campestris* attack and have hypertrophy symptoms, which is abnormal plant outgrowth caused by cell enlargement, at the haustorium attachment sites (Figures 7G,H). The vascular connections between host and parasite, especially the xylem bridges, were enlarged. Based on previous reports, hypertrophy improves the efficiency of root parasitic plant *Phtheirospermum japonicum* parasitism (Spallek et al., 2017). This parasite-derived modification can change host tissue morphology and help with parasite fitness. Similarly, *C. campestris* haustoria not only penetrated and formed vascular connections with *SlNLR* CRISPR tomato vascular tissue, but also changed the overall host stem vascular tissue arrangement, causing a reduction in the secondary xylem in the region of haustorium penetration (Figures 7K,L). This phenotype also indicates that *SlNLR* CRISPR tomato plants are more vulnerable to *C. campestris*.

**FIGURE 8:**
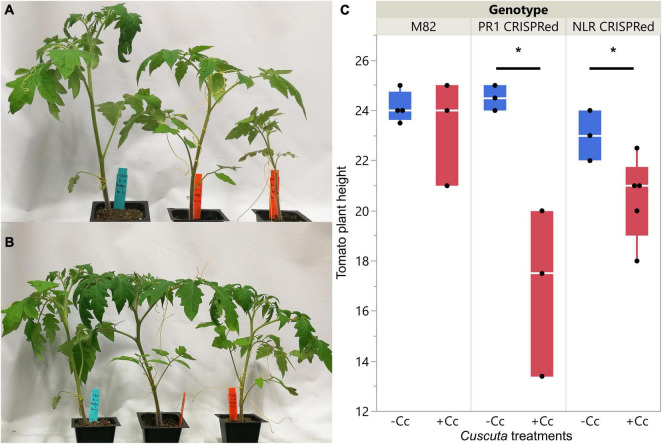
Phenotypes of wild-types and CRISPR transgenic plants with *C. campestris* infestation. **(A)** Wild-type M82 tomatoes and T1 *SlPR1* CRISPR transgenic plants with *C. campestris* infestation. **(B)** Wild-type M82 tomatoes and T1 *SlNLR* CRISPR transgenic plants with *C. campestris* infestation. **(C)** Plant height of wild-type H1706 tomatoes and CRISPR transgenic plants with *C. campestris* infestation. The plant height of host tomato plants was measured in centimeters (cm). Data presented are assessed using one-tailed t-test with wild-type M82 as control. “*” *p*-value < 0.05. *SlPR1* CRISPR transgenic T1 plants #1-3 were used for control (-Cc) and T1 plants #4-6 were used for *Cuscuta* infestation treatments (+ Cc). *SlNLR* CRISPR transgenic T1 plants #1, 5, 9 were used for control (–Cc) and T1 plants #2, 4, 6, 8, 10 were used for *Cuscuta* infestation treatments (+ Cc). Detailed genotyping results of the T1 CRISPR transgenic plants are listed in Supplementary Table 12.

The overall plant phenotypes of wild-type M82 tomatoes and CRISPR transgenic plants with *C. campestris* infestation also showed that the *SlPR1* and *SlNLR* CRISPR transgenic plants have stunted growth after being parasitized by *C. campestris* (Figure 8). The CRISPR transgenic plants with *C. campestris* infestation are much shorter than wild-type M82 with *C. campestris* infestation. Notably, the *SlPR1* and *SlNLR* CRISPR transgenic plants without *C. campestris* infestation have no significant height difference comparing to wild-type M82 (Figure 8C). This result indicates that the CRISPR-mediated mutations do not lead to the stunted growth phenotype directly; however, the *SlPR1* and *SlNLR* knockout mutations cause a growth penalty in the presence of *C. campestris*. These results also suggest that the knockout of candidate genes might interfere with the host defense response and make these CRISPR plants more susceptible to *C. campestris* parasitism.

## Discussion

In this study, we use LCM captured *C. campestris* haustorial tissues and tomato host tissues surrounding haustoria, coupled with RNA-seq analysis to reveal the potential tissue-resolution molecular regulatory machinery and the complexity of gene coexpression networks involved in haustorium organogenesis and host defense responses. We identified three potential key regulators in *C. campestris* that are involved in the early or/and mature stage of haustorium development, and all three of them were validated by using HIGS transgenic plants. We also identified three potential key regulators in tomato plants that are involved in perceiving signals from the parasite, and two of them were further verified with CRISPR knockout mutants.

### Pectin Dynamic Regulation in *Cuscuta parasitism* and Haustorium Development

The chemical structure and mechanical properties of plant cell walls play an important role in organogenesis (Chebli and Geitmann, 2017). Several reports indicate that the physical interactions between pectins and other cell wall components regulate many vital aspects of plant development (Saffer, 2018). Pectin composition and mechanical characteristics have also been found to control the parasitism process and the development of haustorium in *Cuscuta* species. For example, a previous study discovered that *Cuscuta pentagona* secretes de-esterified pectins at the host and parasite interface (Vaughn, 2002). These low-esterified pectins function as a cement to help adhesion to their hosts during the early stage of the *Cuscuta* parasitism process. Similar de-esterified pectin accumulation phenomena have also been reported in *Cuscuta reflexa, Cuscuta campestris*, and *Cuscuta japonica* to facilitate the formation of strong adhesion (Johnsen et al., 2015; Hozumi et al., 2017). De-esterified pectin is a good substrate for pectate lyases, which are also found to be highly expressed at haustoria in *C. reflexa* (Johnsen et al., 2015). High levels of pectate lyases suggest that *Cuscuta* utilizes these enzymes to remodel their host cell walls to achieve successful penetration. The SOM5 and SOM6 GCNs of *C. campestris* genes also identified several highly expressed pectate lyases and pectin methyl-esterases at early and/or mature stages (Figures 2A–C,G–I). Our GCN analysis is not only consistent with previous findings but also provides a more comprehensive potential gene regulatory machinery at specific haustorial developmental stages.

The interplay between PME and PMEI is also known to regulate the chemical and physical characteristics of the cell wall, including cell wall porosity and elasticity (Wormit and Usadel, 2018). Although cell wall loosening is a necessary step for haustorium penetration, an out-of-control cell wall degradation could lead to haustorium detachment from its host (Figure 3). The balance between different pectin enzyme functions might be precisely regulated by many key regulators. The down-regulation of these key regulators might disrupt the dynamic balance between enzymes and lead to abnormal cell wall degradation. Therefore, regulation of the enzymes that likely help with the haustorium penetration process may be disrupted, leading to over-degradation of the host cell wall, resulting in haustorium detachment (Figures 3D–L). Therefore, loosening the host plant cell wall should be precisely regulated during the parasitism process. Several highly expressed PMEIs are found in the SOM3 GCN at the mature stage, verifying this hypothesis (Figures 2D–F). Furthermore, in SOM6 module 1 (Figures 2G–I), tight connections between several enzymes that catalyze the modification of pectin, including PLs, PME, PMT, and PAE, reveal the complexity of dynamic pectin regulation in the haustorium penetration process and the importance of balancing various aspects of cell wall modification.

### Auxin and Ethylene in Haustorium Development

Regulating auxin transport and distribution is a pivotal factor in plant organogenesis (Benková et al., 2003). Regional auxin accumulation is commonly seen in root development, lateral root initiation, and root hair formation. Previous studies also indicate that spatial and temporal auxin accumulation play an important role in the early stage of haustorium organogenesis in root parasitic plants, like *Phtheirospermum japonicum* and *Triphysaria versicolor* (Tomilov et al., 2005; Ishida et al., 2016), which adopted the root morphogenesis program into haustorium development (Yoshida et al., 2016). Our SOM5 and SOM6 GCNs also included several genes that are auxin transporters or auxin-responsive proteins at the early stage of haustorium development (Figure 2). This suggests that auxin-mediated regulation of haustorium initiation might be shared by both root and stem parasitic plants, and also further validates our hypothesis that stem parasites also co-opted the root parasite program into haustorium development.

Other than auxin regulation, ethylene accumulation has also been observed in the early stage of haustorium development in *T. versicolor* (Tomilov et al., 2005). A recent study further discovered that ethylene signaling plays an important role in regulating cell proliferation and differentiation in the haustorial development process of *P. japonicum* (Cui et al., 2020). This root parasitic plant utilizes host-produced ethylene as a signal for host recognition to help with the haustorium penetration process (Cui et al., 2020). However, whether ethylene is also involved in haustorium development in stem parasitic plant remains an open question. The identification of ethylene signaling-related genes in SOM5 GCN (Figure 2C) and *ERF1* as one of the central hub genes in our SOM6 GCN (Figure 2I) provides some clues that ethylene signaling might also play a vital role in regulating haustorium initiation at the early haustorium initiation stage, and later cell differentiation at the mature haustorium stage in *C. campestris*.

### Ethylene and Abscisic Acid in Host Responses Upon *Cuscuta campestris* Infestation

Besides regulating cell wall modification and organogenesis, ethylene is also known as a key hormone involved in plant defense response against various biotic stresses, including pathogens and herbivores (Adie et al., 2007; Liu et al., 2013; Tintor et al., 2013; Böhm et al., 2014). The production of ethylene has often been observed in host plants upon parasitic plant infestation. For example, an ethylene biosynthesis gene (Dos Santos et al., 2003) and an ethylene-responsive element-binding factor (Vieira Dos Santos et al., 2003) were activated in *A. thaliana* upon *O. ramosa* infestation. Similarly, ethylene emission was induced in *N. benthamiana* and *S. lycopersicum* upon *Cuscuta reflexa* infestation *(Hegenauer et al., 2016, 2020).* The identification of many ethylene-responsive transcription factors in our tomato SOM8 and SOM3 GCNs and their tight connections with many LRR and NLR genes (Figure 6) suggest that ethylene may also play a key role in host defense against *C. campestris* by triggering and regulating local and systemic immune responses.

Many previous studies also indicate that ethylene has complex crosstalk with other hormone pathways, including ABA, which is another major phytohormone regulating stress responses (Veselov et al., 2003; Ku et al., 2018; Berens et al., 2019). Although ABA is often known to be involved in responses to abiotic stress (Ku et al., 2018; Berens et al., 2019), induction of ABA biosynthesis and signaling were also observed in the interaction between host and parasitic plants. For example, ABA levels increased in both leaves and roots of tomatoes upon the infestation of root parasitic plant *Phelipanche ramose* (Cheng et al., 2017). ABA concentrations also increased in maize leaves upon *Striga hermonthica* infestation (Taylor et al., 1996). The induction of ABA was also observed in tomatoes at 36 h after *C. pentagona* infestation and continued to accumulate through 120 h (Runyon et al., 2010). Furthermore, in the early stage of *P. ramosa* infestation, elevated gene expression levels of ABA-responsive and biosynthesis genes were also reported. An ABA signaling-related gene has also been included in SOM8 module 2 (Figure 6C) suggests that ABA might play a role in host defense response at the early stage of perceiving *C. campestris* attack.

### Parasitic Plant-Induced Hypertrophy

Hypertrophy, abnormal plant outgrowth caused by cell enlargement, is also a common plant symptom that can be induced by various pathogens, herbivores, or parasites (Bowman, 2019). Hyperplasia is abnormal plant outgrowth caused by excessive cell division, leading to increased cell numbers, resulting in the formation of plant galls that can be induced by viruses, pathogens, parasites, or insects (Bowman, 2019). Parasitic plant-induced hypertrophy and hyperplasia have also been reported in several different systems (Heide-Jørgensen, 2008). For example, hypertrophy has also been observed on crabapple trees, *Malus toringoides*, induced by the stem parasitic plant European mistletoe, *Viscum album (Spallek et al., 2017).* Similarly, the root parasitic plant *Phtheirospermum japonicum* induced hypertrophy at the haustorial attachment site in both *A. thaliana* and tomato roots (Spallek et al., 2017). This hypertrophy phenotype enlarged the width of xylem tissues in the host root right above haustoria attachment sites, which could help the parasites uptake more water and nutrients from the host. The induction of cytokinin and ethylene might be one reason for the hypertrophy phenotype (Spallek et al., 2017; Mignolli et al., 2020; Greifenhagen et al., 2021). However, the detailed mechanism underlying parasitic plant-induced hypertrophy remains unknown.

In this study, our fresh sections showed that *C. campestris* induced xylem bridge cell enlargement in *PR1*-CRISPR transgenic tomato plants (Figure 7). This might allow *C. campestris* to obtain water and nutrients more efficiently from host plants and help with the fitness of the parasite. At the same time, this result also suggests that wild-type tomatoes might originally have a PR1-mediated defense mechanism to prevent hypertrophy upon parasitism. The removal of PR1 makes these transgenic plants more vulnerable to *C. campestris* and these plants have obviously stunted growth upon *C. campestris* infestation (Figures 7, 8). Investigating the connection between PR1 and hypertrophy would be of interest for future research because this could help us to not only understand a potential new defense mechanism against parasitic plants but also know how parasites and host plants influence each other physiologically and morphologically.

### Potential Receptors and Factors Involved in Detecting *Cuscuta campestris* Signals

Detecting pathogens or herbivores is the essential first step in triggering plant innate immunity and the following defense responses. Many plant immune receptors have the leucine-rich repeat (LRR) domain (Padmanabhan et al., 2009). More recent studies have indicated that host plants can identify stem and root parasitic plants by utilizing the mechanisms that are similar to the systems for recognizing bacterial and fungal pathogens. For example, the previously identified *CUSCUTA RECEPTOR 1* (*CuRe1*) encodes a leucine-rich repeat receptor-like protein (LRR-RLP) in tomatoes (Hegenauer et al., 2016). This cell surface receptor-like protein bind with a Cuscuta factor, glycine-rich protein (GRP), or its minimal peptide epitope Crip21 to trigger resistance responses, including hypersensitive responses (HRs) and induced ethylene synthesis (Hegenauer et al., 2016, 2020).

However, the host plants that lack CuRe1 are still fully resistant to *C. reflexa*, indicating that CuRe1 is not the only receptor involved in defense responses against parasitic plants (Hegenauer et al., 2016). Recent studies indicate that multilayered resistance mechanisms are deployed by plants to ensure efficient defense against pathogens and parasites (Jhu et al., 2020). Therefore, investigating other potential receptors is important for identifying other potential layers of defense mechanisms and could help with developing parasitic plant resistant systems in crops. In our LCM RNA-Seq analysis results, many LRR genes, including a CuRe1-like gene, are highly expressed in tomato hosts at the early stage of haustorium penetration and are included in SOM8 module 2 and SOM3 module 3 GCNs (Figure 6). These discoveries not only provide some evidence for the multilayered resistance hypothesis but also identify potential receptors that might be able to perceive different unknown *Cuscuta* signals.

In addition to LRRs, many NLRs encoded by R genes also have been identified to detect parasitic plants. For example, the *RSG3-301* gene encodes a coiled-coil nucleotide-binding site leucine-rich repeat protein (CC-NBS-LRR) protein in the resistant cowpea, which can trigger the hypersensitive response upon *S. gesnerioides* attack (Li and Timko, 2009). Similarly, *Cuscuta R-gene for Lignin-based Resistance 1* (*CuRLR1*) encodes an N-terminal CC-NBS-LRR, which can induce lignin-based resistance responses in the stem cortex of specific resistant Heinz tomato upon *C. campestris* infestation (Jhu et al., 2020). The two NLRs (Solyc07g056200, Solyc12g006040) that are identified in SOM3 module 3 suggest that other NLRs might also be involved in the defense response against *C. campestris*. The tight gene co-expression connection between NLRs and LRRs indicates either that there is potential crosstalk among different layers of resistance mechanisms, or that these receptors might be regulated by common master regulators.

The results of this study reveal the detailed tissue-resolution gene regulatory mechanisms at the parasitic plant and host interface and identifies key regulators of parasitism in both the parasitic plant *C. campestris* and its tomato host. These findings will not only shed light on the field of plant parasitism and haustorium development but also help to develop a parasite-resistant system in tomatoes to reduce economic losses in agriculture. Parasitic weeds-resistant crops will be effective approaches for regulating parasitic plant infestations, reduce the usage of herbicides, and help with developing sustainable agriculture.

## Materials and Methods

### *Cuscuta campestris* Materials

The *C. campestris* plant materials used in this study were generous gifts from W. Thomas Lanini, who collected *C. campestris* seeds from tomato fields in California. By using molecular phylogenetics of plastid DNA, and nuclear large-subunit ribosomal DNA (*nrLSU*) sequences (Stefanović et al., 2007; García et al., 2014; Costea et al., 2015), we have verified that this *Cuscuta* isolate is the same as *Cuscuta campestris* 201, Rose 46281 (WTU) from United States, CA (Jhu et al., 2020) by comparing with published sequences (Costea et al., 2015).

### Haustorium Section Preparation

To capture specific tissues at the host and parasitic plant interface, we prepared haustorium paraffin sections for further analysis. About four-leaf-stage Heinz 1706 tomato (*Solanum lycopersicum*) plants were infested with 10–15 cm long *C. campestris* strands. First, sections of tomato stem with haustoria, about 0.75 cm long, were collected for histology. Second, these stem sections were fixed in formaldehyde – acetic acid – alcohol (FAA). Third, these samples were dehydrated by the ethanol series for one hour at each step (75, 85, 95, 100, 100, and 100% ethanol) and proceeded through xylene in ethanol series for two hours each (25, 50, 75, 100, and 100% xylene). Fourth, these stem sections were then incubated at 42°C in paraffin and xylene solution series and kept in 100% paraffin and changed twice daily for three days at 55°C. Finally, these stem sections were embedded in paraffin (Paraplast X-TRA, Thermo Fisher Scientific). 10 μm thick paraffin sections were prepared using a Leica RM2125RT rotary microtome. These paraffin section strips were placed on polyethylene naphthalate (PEN)-coated membrane slides (Leica), dried at room temperature, and deparaffinized with 100% xylene.

### Laser-Capture Microdissection Sample Collection

Comparing with our previous transcriptome that used the whole tomato stem tissues near the haustorial attachments (Jhu et al., 2020) or the whole *Cuscuta* strands with haustoria (Ranjan et al., 2014) for RNA library construction, to zoom in on the interface between the host and parasite, laser-capture microdissection (LCM) was used for tissue sample collection in this project. With this method, the tissue of *C. campestris* haustorium protruding region and the tomato host tissue that were surrounding the haustoria from paraffin sections were specifically captured for RNA library construction. Targeted haustorial and host tissues were dissected on a Leica LMD6000 Laser Microdissection System (Figure 1). Based on the haustorial structures, we classified haustoria into three different developmental stages. “Early” indicates that the haustorium has just penetrated the tomato stem cortex region. “Intermediate” indicates that the haustorium has penetrated the tomato stem cortex and formed searching hyphae, but has not formed vascular connections with the host vascular system. “Mature” indicates that the haustorium has formed continuous vasculatures with the host (Figure 1). Both haustorial tissues and tomato host tissues were microdissected from each of the three developmental stages. These tissues were collected in lysis buffer from RNAqueous^®^-Micro Total RNA Isolation Kit (Ambion) and stored at −80°C. Collected tissues were processed within one month of fixation to ensure RNA quality. Approximately 30 regions of 10 μm thickness each were cut from each slide, and three to four slides were used per library preparation.

### Laser-Capture Microdissection RNA-Seq Library Preparation and Sequencing

RNAs of these collected tissues were extracted using RNAqueous-Micro Total RNA Isolation Kit (Ambion) and amplified using WT-Ovation Pico RNA Amplification System (ver. 1.0, NuGEN Technologies Inc.) following manufacturer instructions. RNA-seq libraries for Illumina sequencing were constructed following a previously published method (Kumar et al., 2012) with slight modifications. Libraries were quantified, pooled to equal amounts, and their quality was checked on a Bioanalyzer 2100 (Agilent). Libraries were sequenced on a HiSeq2000 Illumina Sequencer at the Vincent J Coates Genomics Sequencing Laboratory at UC Berkeley.

### RNA-Seq Data Mapping and Processing

After receiving raw reads data for these LCM libraries, we separated them into two groups based on tissue origin, *C. campestris* (dodder) and *S. lycopersicum* (tomato). For the LCM RNA-Seq data from *C. campestris*, these raw reads were mapped to the published genome of *C. campestris* (Vogel et al., 2018) with Bowtie 2 (Langmead and Salzberg, 2012). For the LCM RNA-Seq data from tomato, these raw reads were mapped to the published current tomato genome version ITAG4.0 (Sato et al., 2012) with Bowtie 2 (Langmead and Salzberg, 2012). Both data were then analyzed by using EdgeR (Robinson et al., 2009) to get normalized trimmed means of *M*-values (TMM) for further analysis.

### Multidimensional Scaling and Principal Component Analysis With Self-Organizing Maps Clustering

After the normalization steps, to visualize the overall expression profiles of each library, the function “cmdscale” in the R stats package was used to create multidimensional scaling (MDS) data matrix and then generate MDS plots. For both *C. campestris* and tomato LCM RNA-Seq data, genes in the upper 50% quartile of coefficient of variation were selected for further analysis. For principal component analysis (PCA), principal component (PC) values were calculated using the “prcomp” function in the R stats package. The expression profiles of selected genes were visualized in a two-dimensional (2D) plot for PC1 and PC2.

These selected genes were then clustered for multilevel three-by-two hexagonal SOM using the som function in the “kohonen” package (Wehrens and Buydens, 2007). The SOM clustering results were then visualized in PCA plots. The complete gene lists for all SOM units in *C. campestris* and tomato LCM RNA-Seq data with SOM distances and PCA principal component values are included in Supplementary Tables 1, 2, respectively. For both *C. campestris* and tomato LCM RNA-Seq data, we specifically focused on the SOM groups with genes that are highly expressed in the early developmental stage (4 DPA). From *C. campestris* libraries, these genes are likely involved in the mechanisms of haustorium early development and penetration process. From tomato libraries, these genes are likely regulating the early host responses or defense mechanism upon parasitic plant attacks.

### Construction of Gene Co-expression Networks

To identify potential key regulators, we use the genes that are classified in selected SOM groups to build GCNs. The R script is modified from our previously published method (Ichihashi et al., 2014), and the updated script is uploaded to GitHub and included in code availability. For the GCN of *C. campestris* LCM data, we used the selected SOM gene list and constructed the GCN of these genes based on the expression profiles in *C. campestris* LCM data with the following normal quantile cutoffs. The SOM5 GCN cutoff = 0.97. The SOM3 GCN cutoff = 0.98. The SOM6 GCN cutoff = 0.95. For the GCN of tomato LCM data, we used the selected SOM gene list and constructed the GCN of these genes based on the expression profiles in tomato LCM data with the following normal quantile cutoffs. The SOM3 GCN cutoff = 0.80. The SOM8 GCN cutoff = 0.90. These networks were then visualized using Cytoscape version 3.8.0. Based on the number of connections, we identified the central hub genes with the highest connections as our candidate genes (Supplementary Tables 2, 3, 4, 7, 8).

### Functional Annotation and GO Enrichment Analysis of RNA-Seq Data

For tomato genes, the current published tomato genome ITAG4.0 is well-annotated, so the gene name and functional annotation that is currently available on the Sol Genomics Network website (https://solgenomics.net/) were used in this study. For *C. campestris* genes, since many genes are not functionally annotated in the current published *C. campestris* genome (Vogel et al., 2018), we used our previously published master list for annotated *C. campestris* transcriptome (Ranjan et al., 2014) combined with *C. campestris* genome gene IDs to create a more complete functional annotation (Jhu et al., 2021). TAIR ID hits were used for GO Enrichment Analysis on http://geneontology.org/for gene clusters and modules. After obtaining these functional annotations, we specifically focused on our candidate genes that are annotated as transcription factors (TFs) or receptors for further analysis.

### Host-Induced Gene Silencing RNAi and CRISPR Transgenic Plants

For HIGS RNAi constructs for *C. campestris* candidate genes, we used the previously published destination vector pTKO2 vector (Snowden et al., 2005; Brendolise et al., 2017). This pTKOS2 vector contains two GATEWAY cassettes positioned at opposite directions that are separated by an Arabidopsis ACT2 intron, and the whole construct is under the control of the constitutive 35S promoter. To avoid off-target effects on influencing potential homologs in tomatoes, we used BLAST to identify a sequence fragment that is specific to each *C. campestris* candidate gene. We conducted BLASTN analysis with the currently most up-to-date tomato genome ITAG4.0. We were not able to find similar sequences or potential off-target sequences in the tomato genome for our *CcHB7*, *CcPMEI*, and *CcERF1* RNAi constructs. We also conducted BLASTN analysis with the currently available *Cuscuta campestris* genome to ensure no other known genes in the same gene family would be off-target of our RNAi constructs. This was indeed the case except one hit of *CcHB7* RNAi sequence on Cc037848, which is a gene with unknown function. Based on the Blastn results using NCBI database, Cc037848 might be a *HB7-like* gene. However, this gene is only partially similar to *CcHB7* (query coverage percentage is less than 43%) and this *HB7-like* gene was not knocked down by the *CcHB7* RNAi construct (Supplementary Figure 4B).

However, we are also aware that the current *Cuscuta campestris* genome is not as well annotated as the tomato genome. The sequences that are used in HIGS RNAi constructs are listed in Supplementary Table 10. This RNAi fragment was amplified from *C. campestris* genomic DNA and cloned into pCR8/GW-TOPO (Life Technologies), and then *in vitro* recombined with the destination vector pTKO2 to generate a final expression clone. The final plasmids were verified by Sanger sequencing and introduced into *A. tumefaciens* EHA105. Among these RNAi tomato transgenic lines, we did not observe any abnormal growth phenotypes in these RNAi tomato plants grown without *Cuscuta campestris*. Therefore, with all of this information and evidence, we believe that no targets in the host might be affected.

For CRISPR constructs of candidate genes, we identified guide RNA (gRNA) sequences that were specific to the target genes using CCTop - CRISPR/Cas9 target predictor (Stemmer et al., 2015; Labuhn et al., 2017). Among CCTop provided candidates, we identified two sequences that are 50∼150 bp apart at the 5′ of the coding sequence and that are scored highly by the CRISPRscan software (Moreno-Mateos et al., 2015). The gRNA sequences that were used in CRISPR constructs are listed in Supplementary Table 11. One of these two gRNAs was cloned into pDONR_L1R5_U6gRNA and another was cloned into pDONR_L5L2_AtU6-26gRNA. Both plasmids were digested with *Bbs*I, which places gRNAs under a U6 promoter. Using the *in vitro* CRISPR assay, we verified that the selected gRNAs are functional by expressing gRNAs from a T7 promoter (NEB HiScribe T7 High Yield RNA Synthesis Kit E2040S), generating targets by PCR with gene specific primers, and then mixing them with commercial Cas9 protein (NEB *Streptococcus pyogenes* Cas9, M0641S). Next, both gRNA expression cassettes were recombined by multi-site GATEWAY LR cloning into binary plant transformation vector pMR290, in which an Arabidopsis codon-optimized *Streptococcus pyogenes Cas9* is placed under the control of 2 × 35S constitutive promoter. The final plasmids were verified by Sanger sequencing and transformed into *A. tumefaciens* EHA105.

All of these HIGS RNAi and CRISPR constructs were sent to the Ralph M. Parsons Plant Transformation Facility at the University of California Davis to generate transgenic tomato plants. HIGS RNAi transgenic tomato plants are in Heinz 1706 background. To verify that these transgenic plants contained HIGS RNAi constructs, all T0 transgenic plants were selected for kanamycin resistance, and their genomic DNAs were extracted and tested by PCR. CRISPR transgenic tomato plants are in the M82 background. To verify that transgenic plants contained CRISPR mutations in the target gene, a region spanning and extending the regions between the two gRNAs targets were amplified by PCR and sequenced. The sequence results and the mutations generated by CRISPR in T1 plants are shown in Supplementary Figure 7 for each candidate gene.

## Code Availability Statement

The code that are used for RNA-Seq analysis in this study can be found on https://github.com/MinYaoJhu/Cuscuta_LCM_project.

## Data Availability Statement

The datasets presented in this study can be found in online repositories. The names of the repository/repositories and accession number(s) can be found below: https://www.ncbi.nlm.nih.gov/, PRJNA687611 and PRJNA756681.

## Author Contributions

MF conducted LCM to capture tissues and prepared libraries for RNA-Seq and made CRISPR and RNAi constructs and used the UC Davis transformation facility to generate transgenic plants and genotyped T0 transgenic plants. M-YJ mapped the LCM RNA-Seq data to *C. campestris* and tomato genome and performed MDS, PCA, SOM clustering and GO term analysis respectively, constructed gene coexpression networks for selected *C. campestris* and tomato SOM cluster gene lists, analyzed T1 CRISPR and RNAi transgenic plants genotyping and phenotyping data, and wrote the initial manuscript and organized all data to make figures and tables with primary editing from NS. LW performed genotyping and phenotyping on T1 CRISPR and RNAi transgenic plants. KZ conducted qPCR experiments, maintained transgenic tomato seed stocks, and analyzed T1 CRISPR genotyping results. NS supervised this project and serves as the author responsible for contact and communication. All authors contributed to the article and approved the submitted version.

## Conflict of Interest

MF was employed by company The Better Meat Co. The remaining authors declare that the research was conducted in the absence of any commercial or financial relationships that could be construed as a potential conflict of interest.

## Publisher’s Note

All claims expressed in this article are solely those of the authors and do not necessarily represent those of their affiliated organizations, or those of the publisher, the editors and the reviewers. Any product that may be evaluated in this article, or claim that may be made by its manufacturer, is not guaranteed or endorsed by the publisher.

## Supplementary Material

Supplementary Figure 1 Multidimensional scaling (MDS) plot of RNA expression profile in all libraries from LCM of three different *C. campestris* developmental stages mapped to *C. campestris* genome. The early stage has five libraries. The intermediate stage has three libraries. The mature stage has two libraries. This figure is modified from one of the supplemental figures in our previously published paper Jhu et al. (2021).

Supplementary Figure 2 Principal component analysis (PCA) analysis results coupled with self-organizing maps (SOM) clustering in *C. campestris* haustoria across three developmental stages from LCM RNA-Seq data. **(A)** PCA plot of the first and second principal components (PC1 and PC2) and colored to indicate their corresponding SOM groups. Each dot represents a gene. **(B)** Loading values of PC1 and PC2. PC1 separate the mature stage-specific genes from the early and intermediate stage-specific genes. PC2 separates the genes specifically expressed in the early stage from those specifically expressed in the intermediate stage. **(C)** A plot of each SOM group’s scaled expression levels at three *C. campestris* haustorial developmental stages. The color of each line represents the SOM group it belongs to. The 95% confidence interval is indicated by the shaded area around the lines. **(D)** A code plot of SOM clustering showing which developmental phase expressed genes are dominant in each SOM group based on sector size. Each sector represents a developmental stage and is colored according to the stage it represents. **(D,E)** The number 1–6 next to each circle represents the corresponding SOM group. This figure is modified from one of the figures and supplemental figures in our previously published manuscript Jhu et al., 2021 with new information added.

Supplementary Figure 3 Heatmaps and gene-coexpression networks (GCNs) of *C. campestris* haustorial tissues across three developmental stages. **(A)** A heatmap of gene expression profiles in z-scores for SOM1, which includes genes that are highly expressed in the intermediate stage. **(B)** A GCN of genes in SOM1. This SOM1 GCN is composed of five major modules. **(C)** A heatmap of gene expression profiles in z-scores for SOM2, which includes genes that are highly expressed in the intermediate and mature stage. **(D)** A GCN of genes that are in SOM2. The SOM2 GCN is composed of five major modules. **(E)** A heatmap of gene expression profiles in z-scores for SOM4, which includes genes that are relatively highly expressed in the early and intermediate stage. **(F)** A GCN of genes that are in SOM4. The SOM4 GCN is composed of five major modules. The complete gene lists for all SOM units with SOM distances and PCA principal component values are included in Supplementary Table 1. The selected SOM gene lists were used for constructing the GCN based on the expression profiles in *C. campestris* LCM data with the following normal quantile cutoffs. The SOM1 GCN cutoff = 0.97. The SOM2 GCN cutoff = 0.95. The SOM4 GCN cutoff = 0.98.

Supplementary Figure 4 *CcHB7*, *CcHB7-like*, *CcPMEI*, and *CcERF1* expression levels in *C. campestris* haustorium and prehaustorium tissues grown on HIGS transgenic tomatoes. **(A)** Normalized *CcHB7* expression level from qPCR data in *HB7* RNAi transgenic tomatoes. H1706, biological replicates: *n*_*b*_ = 1, technical replicates for each biological replicate: *n*_*t*_ = 4; *HB7* RNAi, biological replicates: *n*_*b*_ = 4, technical replicates for each biological replicate: *n*_*t*_ = 4. **(B)** Normalized *CcHB7-like* expression level from qPCR data in *HB7* RNAi transgenic tomatoes. H1706, biological replicates: *n*_*b*_ = 1, technical replicates for each biological replicate: *n*_*t*_ = 3; *HB7* RNAi, biological replicates: *n*_*b*_ = 4, technical replicates for each biological replicate: *n*_*t*_ = 3 or 4. **(C)** Normalized *CcPMEI* expression level from qPCR data in *PMEI* RNAi transgenic tomatoes. H1706, biological replicates: *n*_*b*_ = 3, technical replicates for each biological replicate: *n*_*t*_ = 4; *PMEI* RNAi, biological replicates: *n*_*b*_ = 5; technical replicates for each biological replicate: *n*_*t*_ = 4. **(D)** Normalized *CcERF1* expression level from qPCR data in *ERF1* RNAi transgenic tomatoes. H1706, biological replicates: *n*_*b*_ = 3, technical replicates for each biological replicate: *n*_*t*_ = 4; *ERF1* RNAi, biological replicates: *n*_*b*_ = 5; technical replicates for each biological replicate: *n*_*t*_ = 4. Data presented are assessed using two-tailed Student’s *t*-test. “*” *p*-value < 0.05. “n.s.” *p*-value > 0.05.

Supplementary Figure 5 Multidimensional scaling (MDS) plot of LCM RNA-Seq data from the host tomato tissues surrounding *C. campestris* haustoria. “Control” means the tomato stem cortex tissue samples that are not next to *C. campestris* haustoria, which serve as negative controls in this experiment. The control has eight libraries. The early stage has eight libraries. The intermediate stage has four libraries. The mature stage has three libraries.

Supplementary Figure 6 Heatmaps and gene-coexpression networks (GCNs) of LCM RNA-Seq data from the host tomato tissues surrounding *C. campestris* haustoria. **(A)** A heatmap of gene expression profiles in z-scores for SOM1, which includes genes specifically highly expressed in the control stage. **(B)** A GCN of genes in SOM1. This SOM1 GCN is composed of five major modules. **(C)** A heatmap of gene expression profiles in z-scores for SOM2, which includes genes specifically highly expressed in the intermediate stage. **(D)** A GCN of genes in SOM2. This SOM2 GCN is composed of five major modules. **(E)** A heatmap of gene expression profiles in *z*-scores for SOM4, which includes genes specifically highly expressed in the control and early stage. **(F)** A GCN of genes in SOM4. This SOM4 GCN is composed of five major modules. **(G)** A heatmap of gene expression profiles in z-scores for SOM5, which includes genes specifically highly expressed in the control and mature stage. **(H)** A GCN of genes in SOM5. This SOM5 GCN is composed of five major modules. **(I)** A heatmap of gene expression profiles in z-scores for SOM6, which includes genes specifically repressed expression in the early stage. **(J)** A GCN of genes in SOM6. This SOM6 GCN is composed of five major modules. **(K)** A heatmap of gene expression profiles in *z*-scores for SOM7, which includes genes specifically repressed expression in the mature stage. **(L)** A GCN of genes in SOM7. This SOM7 GCN is composed of five major modules. The complete gene lists for all SOM units with SOM distances and PCA principal component values are included in Supplementary Table 6. The selected SOM gene lists were used for constructing the GCN based on the expression profiles in tomato LCM data with the following normal quantile cutoffs. The SOM1 GCN cutoff = 0.95. The SOM2 GCN cutoff = 0.93. The SOM4 GCN cutoff = 0.93. The SOM5 GCN cutoff = 0.96. The SOM6 GCN cutoff = 0.94. The SOM7 GCN cutoff = 0.92.

Supplementary Figure 7 Genotyping results of the T1 CRISPR transgenic plants that are used in this study. **(A)** Genomic DNA PCR product sequencing result of PR1 T1 CRISPR plants. T1 plant #1-3 have similar mixed peaks were present next to the sgRNA1, indicating a biallelic or heterozygous mutation. **(B)** Genomic DNA PCR product sequencing result of NLR T1 CRISPR plants. T1 plant #1, 2, 6 have similar mixed peaks were present next to the sgRNA1, indicating a biallelic or heterozygous mutation. T1 plant #4 might have a large section of deletion (> 150 bp) next to the two gRNAs, so it has a large section of mismatch and mixed peaks. **(A,B)** The dark red line indicates the sequencing result. The number next to each dark red line indicates the T1 plant ID number (T1 plant #). The section filled with dark red color indicates regions of perfect sequence match; the empty boxes indicate regions of sequence mismatch. The dark red arrowhead above each sequence indicates a section of insertion. The dark red line above each sequence indicates a single base pair insertion. The gRNAs are labeled as blue arrows. The gRNA sequences that were used in these CRISPR constructs are listed in Supplementary Table 11. The protospacer adjacent motif (PAM) sites are labeled as small red boxes that are right next to gRNAs. Detailed genotyping results of the T1 CRISPR transgenic plants are listed in Supplementary Table 12.

## References

[B1] Adie B. Chico J. M. Rubio-Somoza I. Solano R. (2007). Modulation of Plant Defenses by Ethylene. *J. Plant Growth Regul.* 26 160–177.

[B2] Agrios G. N. (2005). *Chapter Thirteen - Plant Diseases Caused by Parasitic Higher Plants, Invasive Climbing Plants, and Parasitic Green Algae.* San Diego: Academic Press. 705–722.

[B3] Alakonya A. Kumar R. Koenig D. Kimura S. Townsley B. Runo S. (2012). Interspecific RNA Interference of SHOOT MERISTEMLESS-Like Disrupts Cuscuta pentagona Plant Parasitism. *Plant Cell* 24 3153–3166. 10.1105/tpc.112.099994 22822208 PMC3426138

[B4] Benková E. Michniewicz M. Sauer M. Teichmann T. Seifertová D. Jürgens G. (2003). Local, efflux-dependent auxin gradients as a common module for plant organ formation. *Cell* 115 591–602. 10.1016/s0092-8674(03)00924-3 14651850

[B5] Berens M. L. Wolinska K. W. Spaepen S. Ziegler J. Nobori T. Nair A. (2019). Balancing trade-offs between biotic and abiotic stress responses through leaf age-dependent variation in stress hormone cross-talk. *Proc. Natl. Acad. Sci.* 116 2364–2373. 10.1073/pnas.1817233116 30674663 PMC6369802

[B6] Böhm H. Albert I. Fan L. Reinhard A. Nürnberger T. (2014). Immune receptor complexes at the plant cell surface. *Curr. Opin. Plant Biol.* 20 47–54. 10.1016/j.pbi.2014.04.007 24835204

[B7] Bowman C. (2019). *Plant Virology.* United Kingdom: Edtech.

[B8] Breen S. Williams S. J. Outram M. Kobe B. Solomon P. S. (2017). Emerging Insights into the Functions of Pathogenesis-Related Protein 1. *Trends Plant Sci.* 22 871–879. 10.1016/j.tplants.2017.06.013 28743380

[B9] Brendolise C. Montefiori M. Dinis R. Peeters N. Storey R. D. Rikkerink E. H. (2017). A novel hairpin library-based approach to identify NBS-LRR genes required for effector-triggered hypersensitive response in *Nicotiana benthamiana*. *Plant Methods.* 13:32. 10.1186/s13007-017-0181-7 PMC5408436 28465712

[B10] Broekgaarden C. Caarls L. Vos I. A. Pieterse C. M. J. Van Wees S. C. M. (2015). Ethylene: traffic Controller on Hormonal Crossroads to Defense. *Plant Physiol.* 169 2371–2379. 10.1104/pp.15.01020 26482888 PMC4677896

[B11] Chebli Y. Geitmann A. (2017). Cellular growth in plants requires regulation of cell wall biochemistry. *Curr. Opin. Cell Biol.* 44 28–35. 10.1016/j.ceb.2017.01.002 28131101

[B12] Cheng X. Floková K. Bouwmeester H. Ruyter-Spira C. (2017). The Role of Endogenous Strigolactones and Their Interaction with ABA during the Infection Process of the Parasitic Weed Phelipanche ramosa in Tomato Plants. *Front. Plant Sci.* 8:392. 10.3389/fpls.2017.00392 28392795 PMC5364151

[B13] Clauset A. Newman M. E. J. Moore C. (2004). Finding community structure in very large networks. *Phys. Rev. E* 70:066111. 10.1103/PhysRevE.70.066111 15697438

[B14] Cline M. S. Smoot M. Cerami E. Kuchinsky A. Landys N. Workman C. (2007). Integration of biological networks and gene expression data using Cytoscape. *Nat. Protocols* 2 2366–2382. 10.1038/nprot.2007.324 17947979 PMC3685583

[B15] Costea M. García M. A. Baute K. Stefanović S. (2015). Entangled evolutionary history of Cuscuta pentagona clade: a story involving hybridization and Darwin in the Galapagos. *TAXON* 64 1225–1242.

[B16] Cui S. Kubota T. Nishiyama T. Ishida J. K. Shigenobu S. Shibata T. F. (2020). Ethylene signaling mediates host invasion by parasitic plants. *Sci. Adv.* 6:eabc2385. 10.1126/sciadv.abc2385 33115743 PMC7608805

[B17] Dos Santos C. V. Letousey P. Delavault P. Thalouarn P. (2003). Defense Gene Expression Analysis of Arabidopsis thaliana Parasitized by Orobanche ramosa. *Phytopathology* 93 451–457. 10.1094/PHYTO.2003.93.4.451 18944360

[B18] Fishman M. R. Shirasu K. (2021). How to resist parasitic plants: pre- and post-attachment strategies. *Curr. Opin. Plant Biol.* 62:102004. 10.1016/j.pbi.2021.102004 33647828

[B19] Fürst U. Hegenauer V. Kaiser B. Körner M. Welz M. Albert M. (2016). Parasitic Cuscuta factor(s) and the detection by tomato initiates plant defense. *Commun. Integr. Biol.* 9:e1244590. 10.1080/19420889.2016.1244590 28042379 PMC5193051

[B20] Furuhashi T. Furuhashi K. Weckwerth W. (2011). The parasitic mechanism of the holostemparasitic plant Cuscuta. *J. Plant Interact.* 6 207–219.

[B21] García M. A. Costea M. Kuzmina M. Stefanović S. (2014). Phylogeny, character evolution, and biogeography of Cuscuta (dodders; Convolvulaceae) inferred from coding plastid and nuclear sequences. *Am. J. Bot.* 101 670–690. 10.3732/ajb.1300449 24688058

[B22] Greifenhagen A. Braunstein I. Pfannstiel J. Yoshida S. Shirasu K. Schaller A. (2021). The Phtheirospermum japonicum isopentenyltransferase PjIPT1a regulates host cytokinin responses in Arabidopsis. *New Phytol.* 232 1582–1590. 10.1111/nph.17615 34254310

[B23] Hegenauer V. Fürst U. Kaiser B. Smoker M. Zipfel C. Felix G. (2016). Detection of the plant parasite Cuscuta reflexa by a tomato cell surface receptor. *Science* 353 478–481. 10.1126/science.aaf3919 27471302

[B24] Hegenauer V. Slaby P. Körner M. Bruckmüller J.-A. Burggraf R. Albert I. (2020). The tomato receptor CuRe1 senses a cell wall protein to identify Cuscuta as a pathogen. *Nat. Commun.* 11:5299. 10.1038/s41467-020-19147-4 33082345 PMC7576778

[B25] Heide-Jørgensen H. (2008). *Parasitic Flowering Plants.* Netherlands: Brill.

[B26] Hozumi A. Bera S. Fujiwara D. Obayashi T. Yokoyama R. Nishitani K. (2017). Arabinogalactan Proteins Accumulate in the Cell Walls of Searching Hyphae of the Stem Parasitic Plants, Cuscuta campestris and Cuscuta japonica. *Plant Cell Physiol.* 58 1868–1877. 10.1093/pcp/pcx121 29016904

[B27] Ichihashi Y. Aguilar-Martínez J. A. Farhi M. Chitwood D. H. Kumar R. Millon L. V. (2014). Evolutionary developmental transcriptomics reveals a gene network module regulating interspecific diversity in plant leaf shape. *Proc. Natl. Acad. Sci.* 111 E2616–E2621. 10.1073/pnas.1402835111 24927584 PMC4078850

[B28] Ishida J. K. Wakatake T. Yoshida S. Takebayashi Y. Kasahara H. Wafula E. (2016). Local Auxin Biosynthesis Mediated by a YUCCA Flavin Monooxygenase Regulates Haustorium Development in the Parasitic Plant Phtheirospermum japonicum. *Plant Cell* 28 1795–1814. 10.1105/tpc.16.00310 27385817 PMC5006708

[B29] Jhu M.-Y. Farhi M. Wang L. Philbrook R. N. Belcher M. S. Nakayama H. (2020). Lignin-based resistance to Cuscuta campestris parasitism in Heinz resistant tomato cultivars. *bioRxiv* 706861. 10.1101/706861 PMC9070836 35099559

[B30] Jhu M.-Y. Ichihashi Y. Farhi M. Wong C. Sinha N. R. (2021). LATERAL ORGAN BOUNDARIES DOMAIN 25 functions as a key regulator of haustorium development in dodders. *Plant Physiol.* 186 2093–2110. 10.1093/plphys/kiab231 34618110 PMC8331169

[B31] Johnsen H. R. Striberny B. Olsen S. Vidal-Melgosa S. Fangel J. U. Willats W. G. T. (2015). Cell wall composition profiling of parasitic giant dodder (Cuscuta reflexa) and its hosts: a priori differences and induced changes. *New Phytol.* 207 805–816. 10.1111/nph.13378 25808919

[B32] Johnson N. R. Depamphilis C. W. Axtell M. J. (2019). Compensatory sequence variation between trans-species small RNAs and their target sites. *Elife* 8:e49750. 10.7554/eLife.49750 31845648 PMC6917502

[B33] Kim G. Leblanc M. L. Wafula E. K. Depamphilis C. W. Westwood J. H. (2014). Genomic-scale exchange of mRNA between a parasitic plant and its hosts. *Science* 345 808–811. 10.1126/science.1253122 25124438

[B34] Kimura S. Sinha N. (2008). Tomato (Solanum lycopersicum): a Model Fruit-Bearing Crop. *Cold Spring Harbor Protocols* 2008:db.emo105. 10.1101/pdb.emo105 21356708

[B35] Kokla A. Leso M. Zhang X. Simura J. Cui S. Ljung K. (2021). Nitrates increase abscisic acid levels to regulate haustoria formation in the parasitic plant Phtheirospermum japonicum. *Biorxiv* 2021. 10.1101/2021.06.15.448499 PMC9142502 35624089

[B36] Kong G. Wan L. Deng Y. Z. Yang W. Li W. Jiang L. (2019). Pectin acetylesterase PAE5 is associated with the virulence of plant pathogenic oomycete Peronophythora litchii. *Physiol. Mol. Plant Pathol.* 106 16–22.

[B37] Ku Y.-S. Sintaha M. Cheung M.-Y. Lam H.-M. (2018). Plant Hormone Signaling Crosstalks between Biotic and Abiotic Stress Responses. *Int. J. Mol. Sci.* 19:3206. 10.3390/ijms19103206 30336563 PMC6214094

[B38] Kumar R. Ichihashi Y. Kimura S. Chitwood D. Headland L. Peng J. (2012). A High-Throughput Method for Illumina RNA-Seq Library Preparation. *Front. Plant Sci.* 3:202. 10.3389/fpls.2012.00202 22973283 PMC3428589

[B39] Labuhn M. Adams F. F. Ng M. Knoess S. Schambach A. Charpentier E. M. (2017). Refined sgRNA efficacy prediction improves large- and small-scale CRISPR–Cas9 applications. *Nucleic Acids Res.* 46 1375–1385. 10.1093/nar/gkx1268 29267886 PMC5814880

[B40] Langmead B. Salzberg S. L. (2012). Fast gapped-read alignment with Bowtie 2. *Nat. Methods* 9 357–359. 10.1038/nmeth.1923 22388286 PMC3322381

[B41] Lanini W. Kogan M. (2005). Biology and Management of Cuscuta in Crops. *Ciencia Investig. Agrar.* 32 127–141. 10.1371/journal.pone.0081389 24312295 PMC3842250

[B42] Li J. Timko M. P. (2009). Gene-for-Gene Resistance in Striga-Cowpea Associations. *Science* 325 1094–1094. 10.1126/science.1174754 19713520

[B43] Liu Z. Wu Y. Yang F. Zhang Y. Chen S. Xie Q. (2013). BIK1 interacts with PEPRs to mediate ethylene-induced immunity. *Proc. Natl. Acad. Sci.* 110 6205–6210. 10.1073/pnas.1215543110 23431184 PMC3625333

[B44] McHale L. Tan X. Koehl P. Michelmore R. W. (2006). Plant NBS-LRR proteins: adaptable guards. *Genome Biol.* 7:212. 10.1186/gb-2006-7-4-212 16677430 PMC1557992

[B45] Mignolli F. Todaro J. S. Vidoz M. L. (2020). Internal aeration and respiration of submerged tomato hypocotyls are enhanced by ethylene-mediated aerenchyma formation and hypertrophy. *Physiol. Plantarum* 169 49–63. 10.1111/ppl.13044 31688957

[B46] Moreno-Mateos M. A. Vejnar C. E. Beaudoin J.-D. Fernandez J. P. Mis E. K. Khokha M. K. (2015). CRISPRscan: designing highly efficient sgRNAs for CRISPR-Cas9 targeting *in vivo*. *Nat. Methods* 12 982–988. 10.1038/nmeth.3543 26322839 PMC4589495

[B47] O’Brien T. P. Feder N. Mccully M. E. (1964). Polychromatic staining of plant cell walls by toluidine blue O. *Protoplasma* 59 368–373. 10.1007/bf01248568

[B48] Padmanabhan M. Cournoyer P. Dinesh-Kumar S. P. (2009). The leucine-rich repeat domain in plant innate immunity: a wealth of possibilities. *Cell. Microbiol.* 11 191–198. 10.1111/j.1462-5822.2008.01260.x 19016785 PMC2762402

[B49] Pan C. Ye L. Qin L. Liu X. He Y. Wang J. (2016). CRISPR/Cas9-mediated Efficient and Heritable Targeted Mutagenesis in Tomato Plants in the First and Later Generations. *Sci. Rep.* 6:24765. 10.1038/srep24765 PMC4838866 27097775

[B50] Pehlivan N. (2019). Stochasticity in transcriptional expression of a negative regulator of Arabidopsis ABA network. *3 Biotech* 9:15. 10.1007/s13205-018-1542-2 30622853 PMC6314942

[B51] Ranjan A. Ichihashi Y. Farhi M. Zumstein K. Townsley B. David-Schwartz R. (2014). De Novo Assembly and Characterization of the Transcriptome of the Parasitic Weed Dodder Identifies Genes Associated with Plant Parasitism. *Plant Physiol.* 166 1186–1199. 10.1104/pp.113.234864 24399359 PMC4226353

[B52] Robinson M. D. Mccarthy D. J. Smyth G. K. (2009). edgeR: a Bioconductor package for differential expression analysis of digital gene expression data. *Bioinformatics* 26 139–140. 10.1093/bioinformatics/btp616 19910308 PMC2796818

[B53] Runyon J. B. Mescher M. C. Felton G. W. De Moraes C. M. (2010). Parasitism by Cuscuta pentagona sequentially induces JA and SA defence pathways in tomato. *Plant Cell Environ.* 33 290–303. 10.1111/j.1365-3040.2009.02082.x 19930126

[B54] Saffer A. M. (2018). Expanding roles for pectins in plant development. *J. Integr. Plant Biol.* 60 910–923. 10.1111/jipb.12662 29727062

[B55] Sato S. Tabata S. Hirakawa H. Asamizu E. Shirasawa K. Isobe S. (2012). The tomato genome sequence provides insights into fleshy fruit evolution. *Nature* 485 635–641. 10.1038/nature11119 22660326 PMC3378239

[B56] Shen G. Liu N. Zhang J. Xu Y. Baldwin I. T. Wu J. (2020). Cuscuta australis (dodder) parasite eavesdrops on the host plants’ FT signals to flower. *Proc. Natl. Acad. Sci.* 117 23125–23130. 10.1073/pnas.2009445117 32868415 PMC7502711

[B57] Shimizu K. Aoki K. (2019). Development of Parasitic Organs of a Stem Holoparasitic Plant in Genus Cuscuta. *Front. Plant Sci.* 10:1435. 10.3389/fpls.2019.01435 31781146 PMC6861301

[B58] Snowden K. Simkin A. Janssen B. Templeton K. Loucas H. Simons J. (2005). The decreased apical dominance1/Petunia hybrida CAROTENOID CLEAVAGE DIOXYGENASE8 gene affects branch production and plays a role in leaf senescence, root growth, and flower development. *Plant Cell.* 17 746–759. 10.1105/tpc.104.027714. 15705953 PMC1069696

[B59] Spallek T. Melnyk C. W. Wakatake T. Zhang J. Sakamoto Y. Kiba T. (2017). Interspecies hormonal control of host root morphology by parasitic plants. *Proc. Natl. Acad. Sci.* 114 5283–5288. 10.1073/pnas.1619078114 28461500 PMC5441792

[B60] Stefanović S. Kuzmina M. Costea M. (2007). Delimitation of major lineages within Cuscuta subgenus Grammica (Convolvulaceae) using plastid and nuclear DNA sequences. *Am. J. Bot.* 94 568–589. 10.3732/ajb.94.4.568 21636427

[B61] Stemmer M. Thumberger T. Del Sol Keyer M. Wittbrodt J. Mateo J. L. (2015). CCTop: an Intuitive, Flexible and Reliable CRISPR/Cas9 Target Prediction Tool. *Plos One* 10:e0124633. 10.1371/journal.pone.0124633 25909470 PMC4409221

[B62] Su C. Liu H. Wafula E. K. Honaas L. De Pamphilis C. W. Timko M. P. (2020). SHR4z, a novel decoy effector from the haustorium of the parasitic weed Striga gesnerioides, suppresses host plant immunity. *New Phytol.* 226 891–908. 10.1111/nph.16351 31788811 PMC7187149

[B63] Tada Y. Sugai M. Furuhashi K. (1996). Haustoria of Cuscuta japonica, a Holoparasitic Flowering Plant, Are Induced by the Cooperative Effects of Far-Red Light and Tactile Stimuli. *Plant Cell Physiol.* 37 1049–1053. 10.1093/pcp/pcd070 11134423

[B64] Taylor A. Martin J. Seel W. E. (1996). Physiology of the parasitic association between maize and witchweed (Striga hermonthica): is ABA involved? *J. Exp. Bot.* 47 1057–1065. 10.1093/jxb/47.8.1057 12432039

[B65] Tintor N. Ross A. Kanehara K. Yamada K. Fan L. Kemmerling B. (2013). Layered pattern receptor signaling *via* ethylene and endogenous elicitor peptides during Arabidopsis immunity to bacterial infection. *Proc. Natl. Acad. Sci.* 110 6211–6216. 10.1073/pnas.1216780110 23431187 PMC3625345

[B66] Tomilov A. A. Tomilova N. B. Abdallah I. Yoder J. I. (2005). Localized hormone fluxes and early haustorium development in the hemiparasitic plant Triphysaria versicolor. *Plant Physiol.* 138 1469–1480. 10.1104/pp.104.057836 15965023 PMC1176418

[B67] Valdés A. E. övernäs E. Johansson H. Rada-Iglesias A. Engström P. (2012). The homeodomain-leucine zipper (HD-Zip) class I transcription factors ATHB7 and ATHB12 modulate abscisic acid signalling by regulating protein phosphatase 2C and abscisic acid receptor gene activities. *Plant Mol. Biol.* 80 405–418. 10.1007/s11103-012-9956-4 22968620

[B68] Van Ghelder C. Parent G. J. Rigault P. Prunier J. Giguère I. Caron S. (2019). The large repertoire of conifer NLR resistance genes includes drought responsive and highly diversified RNLs. *Sci. Rep.* 9:11614. 10.1038/s41598-019-47950-7 31406137 PMC6691002

[B69] Vaughn K. C. (2002). Attachment of the parasitic weed dodder to the host. *Protoplasma* 219 227–237. 10.1007/s007090200024 12099223

[B70] Veselov D. Langhans M. Hartung W. Aloni R. Feussner I. Götz C. (2003). Development of *Agrobacterium tumefaciens* C58-induced plant tumors and impact on host shoots are controlled by a cascade of jasmonic acid, auxin, cytokinin, ethylene and abscisic acid. *Planta* 216 512–522. 10.1007/s00425-002-0883-5 12520344

[B71] Vieira Dos Santos C. Delavault P. Letousey P. Thalouarn P. (2003). Identification by suppression subtractive hybridization and expression analysis of Arabidopsis thaliana putative defence genes during Orobanche ramosa infection. *Physiol. Mol. Plant Pathol.* 62 297–303. 10.1016/s0885-5765(03)00073-0

[B72] Vogel A. Schwacke R. Denton A. K. Usadel B. Hollmann J. Fischer K. (2018). Footprints of parasitism in the genome of the parasitic flowering plant Cuscuta campestris. *Nat. Commun.* 9:2515. 10.1038/s41467-018-04344-z 29955043 PMC6023873

[B73] Wehrens R. Buydens L. (2007). Self- and Super-organizing Maps in R: the kohonen Package. *J. Statist. Softw.* 21 1–19.

[B74] Wormit A. Usadel B. (2018). The Multifaceted Role of Pectin Methylesterase Inhibitors (PMEIs). *Int. J. Mol. Sci.* 19:2878. 10.3390/ijms19102878 30248977 PMC6213510

[B75] Yaakov G. Lanini W. T. Wrobel R. L. (2001). Tolerance of Tomato Varieties to Lespedeza Dodder. *Weed Sci.* 49 520–523.

[B76] Yoder J. I. Scholes J. D. (2010). Host plant resistance to parasitic weeds; recent progress and bottlenecks. *Curr. Opin. Plant Biol.* 13 478–484. 10.1016/j.pbi.2010.04.011 20627804

[B77] Yoshida S. Cui S. Ichihashi Y. Shirasu K. (2016). The Haustorium, a Specialized Invasive Organ in Parasitic Plants. *Annu. Rev. Plant Biol.* 67 643–667. 10.1146/annurev-arplant-043015-111702 27128469

