# Supplementary figures and images for: Investigating Host and Parasitic Plant Interaction by Tissue-Specific Gene Analyses on Tomato and Cuscuta campestris Interface at Three Haustorial Developmental Stages
Source: Front Plant Sci. 2022 Feb 10;12:764843. doi: 10.3389/fpls.2021.764843 (PMC8866705; doi:10.3389/fpls.2021.764843)

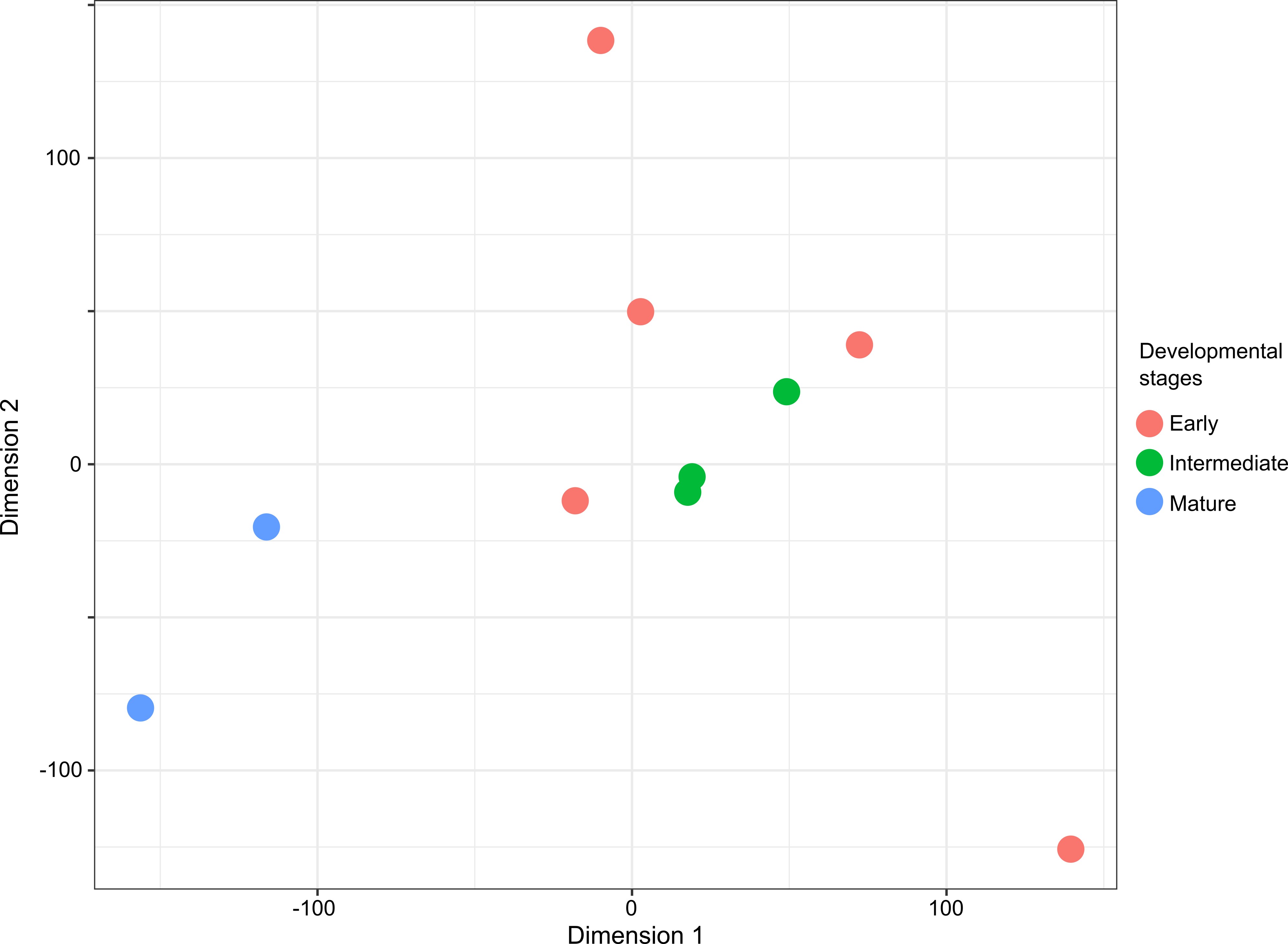

Supplement: Supplementary Figure 1 — Multidimensional scaling (MDS) plot of RNA expression profile in all libraries from LCM of three different C. campestris developmental stages mapped to C. campestris genome. The early stage has five libraries. The intermediate stage has three libraries. The mature stage has two libraries. This figure is modified from one of the supplemental figures in our previously published paper Jhu et al. (2021). [file Image_1.JPEG]

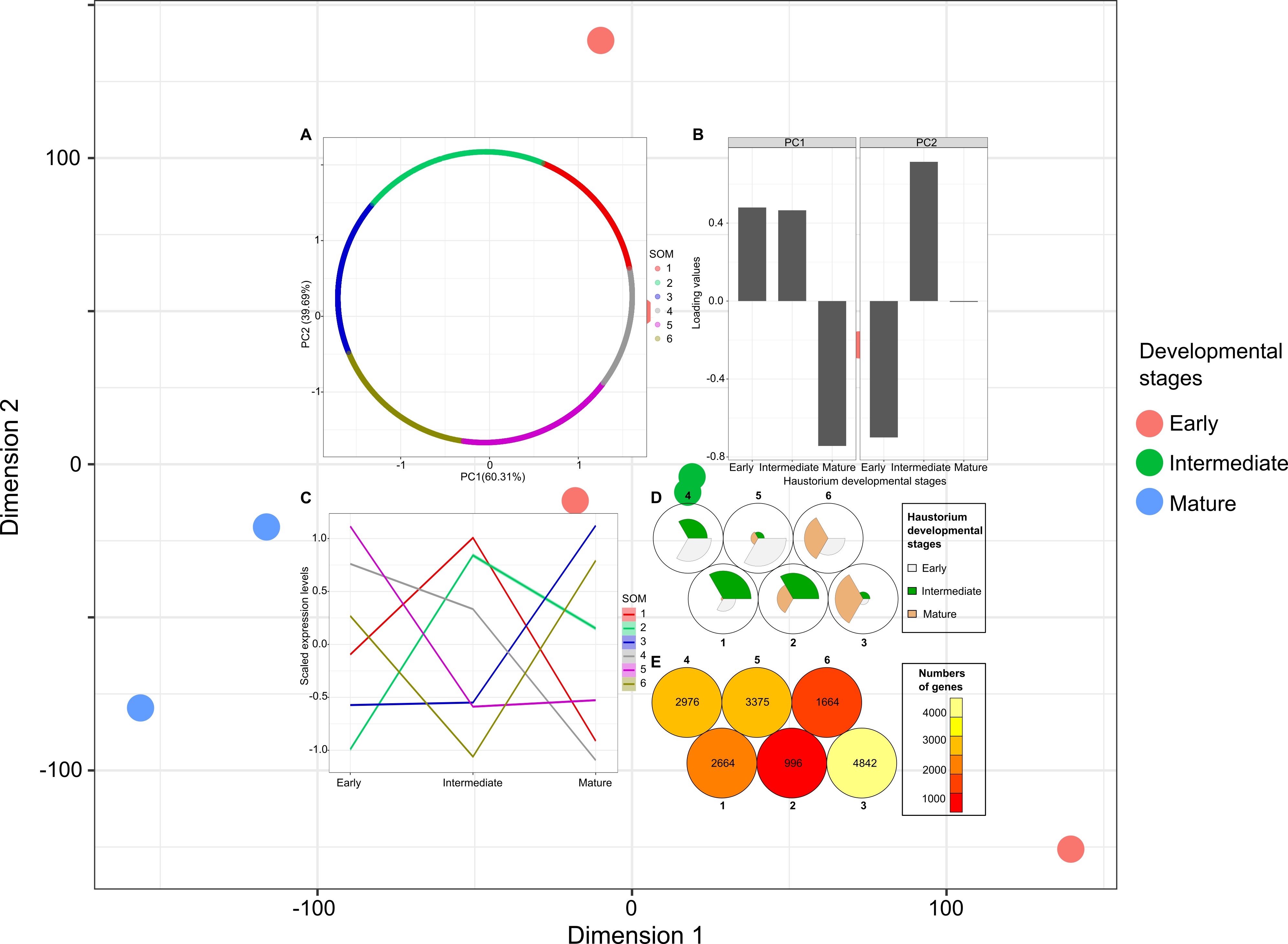

Supplement: Supplementary Figure 2 — Principal component analysis (PCA) analysis results coupled with self-organizing maps (SOM) clustering in C. campestris haustoria across three developmental stages from LCM RNA-Seq data. (A) PCA plot of the first and second principal components (PC1 and PC2) and colored to indicate their corresponding SOM groups. Each dot represents a gene. (B) Loading values of PC1 and PC2. PC1 separate the mature stage-specific genes from the early and intermediate stage-specific genes. PC2 separates the genes specifically expressed in the early stage from those specifically expressed in the intermediate stage. (C) A plot of each SOM group’s scaled expression levels at three C. campestris haustorial developmental stages. The color of each line represents the SOM group it belongs to. The 95% confidence interval is indicated by the shaded area around the lines. (D) A code plot of SOM clustering showing which developmental phase expressed genes are dominant in each SOM group based on sector size. Each sector represents a developmental stage and is colored according to the stage it represents. (D,E) The number 1–6 next to each circle represents the corresponding SOM group. This figure is modified from one of the figures and supplemental figures in our previously published manuscript Jhu et al., 2021 with new information added. [file Image_2.JPEG]

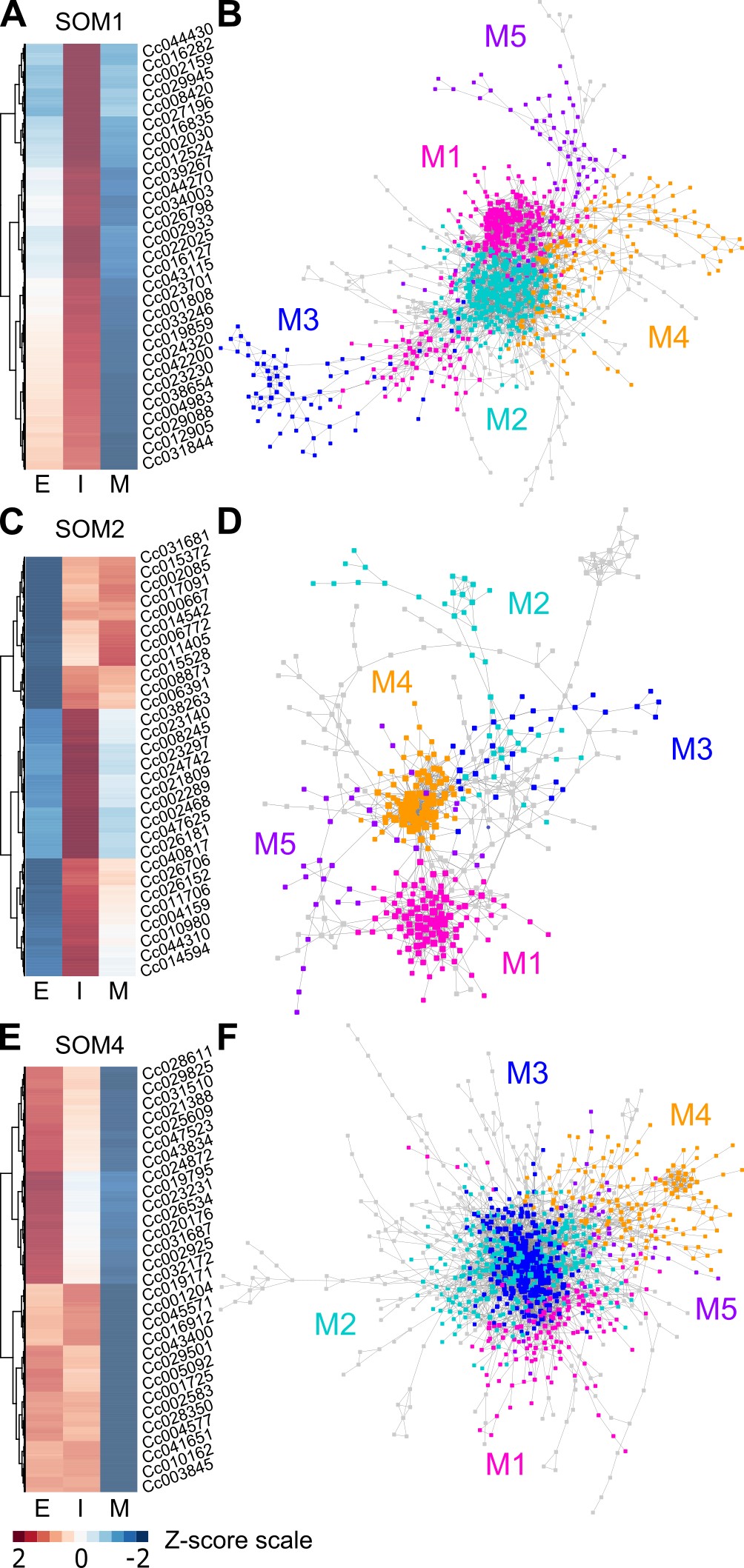

Supplement: Supplementary Figure 3 — Heatmaps and gene-coexpression networks (GCNs) of C. campestris haustorial tissues across three developmental stages. (A) A heatmap of gene expression profiles in z-scores for SOM1, which includes genes that are highly expressed in the intermediate stage. (B) A GCN of genes in SOM1. This SOM1 GCN is composed of five major modules. (C) A heatmap of gene expression profiles in z-scores for SOM2, which includes genes that are highly expressed in the intermediate and mature stage. (D) A GCN of genes that are in SOM2. The SOM2 GCN is composed of five major modules. (E) A heatmap of gene expression profiles in z-scores for SOM4, which includes genes that are relatively highly expressed in the early and intermediate stage. (F) A GCN of genes that are in SOM4. The SOM4 GCN is composed of five major modules. The complete gene lists for all SOM units with SOM distances and PCA principal component values are included in Supplementary Table 1. The selected SOM gene lists were used for constructing the GCN based on the expression profiles in C. campestris LCM data with the following normal quantile cutoffs. The SOM1 GCN cutoff = 0.97. The SOM2 GCN cutoff = 0.95. The SOM4 GCN cutoff = 0.98. [file Image_3.JPEG]

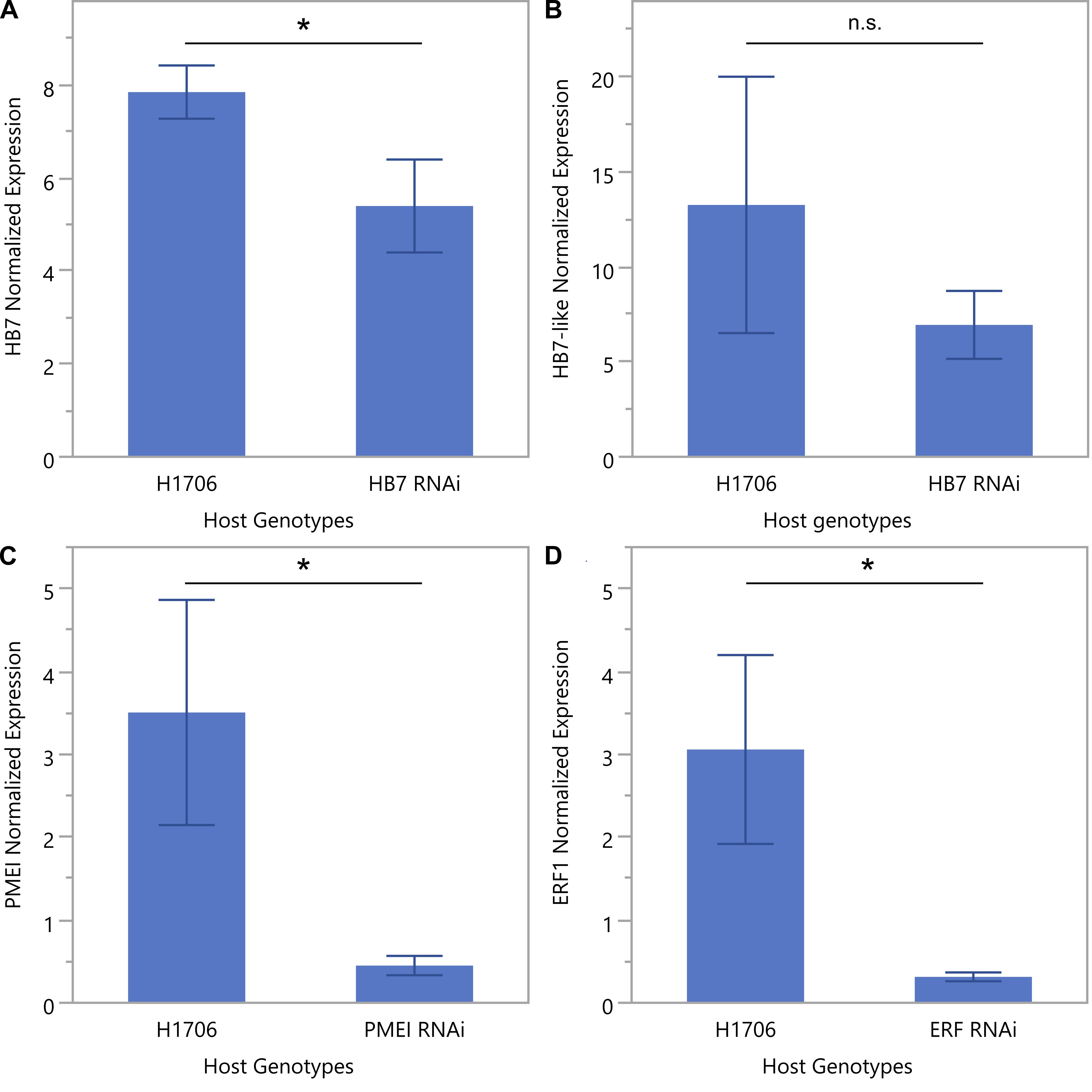

Supplement: Supplementary Figure 4 — CcHB7, CcHB7-like, CcPMEI, and CcERF1 expression levels in C. campestris haustorium and prehaustorium tissues grown on HIGS transgenic tomatoes. (A) Normalized CcHB7 expression level from qPCR data in HB7 RNAi transgenic tomatoes. H1706, biological replicates: nb = 1, technical replicates for each biological replicate: nt = 4; HB7 RNAi, biological replicates: nb = 4, technical replicates for each biological replicate: nt = 4. (B) Normalized CcHB7-like expression level from qPCR data in HB7 RNAi transgenic tomatoes. H1706, biological replicates: nb = 1, technical replicates for each biological replicate: nt = 3; HB7 RNAi, biological replicates: nb = 4, technical replicates for each biological replicate: nt = 3 or 4. (C) Normalized CcPMEI expression level from qPCR data in PMEI RNAi transgenic tomatoes. H1706, biological replicates: nb = 3, technical replicates for each biological replicate: nt = 4; PMEI RNAi, biological replicates: nb = 5; technical replicates for each biological replicate: nt = 4. (D) Normalized CcERF1 expression level from qPCR data in ERF1 RNAi transgenic tomatoes. H1706, biological replicates: nb = 3, technical replicates for each biological replicate: nt = 4; ERF1 RNAi, biological replicates: nb = 5; technical replicates for each biological replicate: nt = 4. Data presented are assessed using two-tailed Student’s t-test. “*” p-value < 0.05. “n.s.” p-value > 0.05. [file Image_4.JPEG]

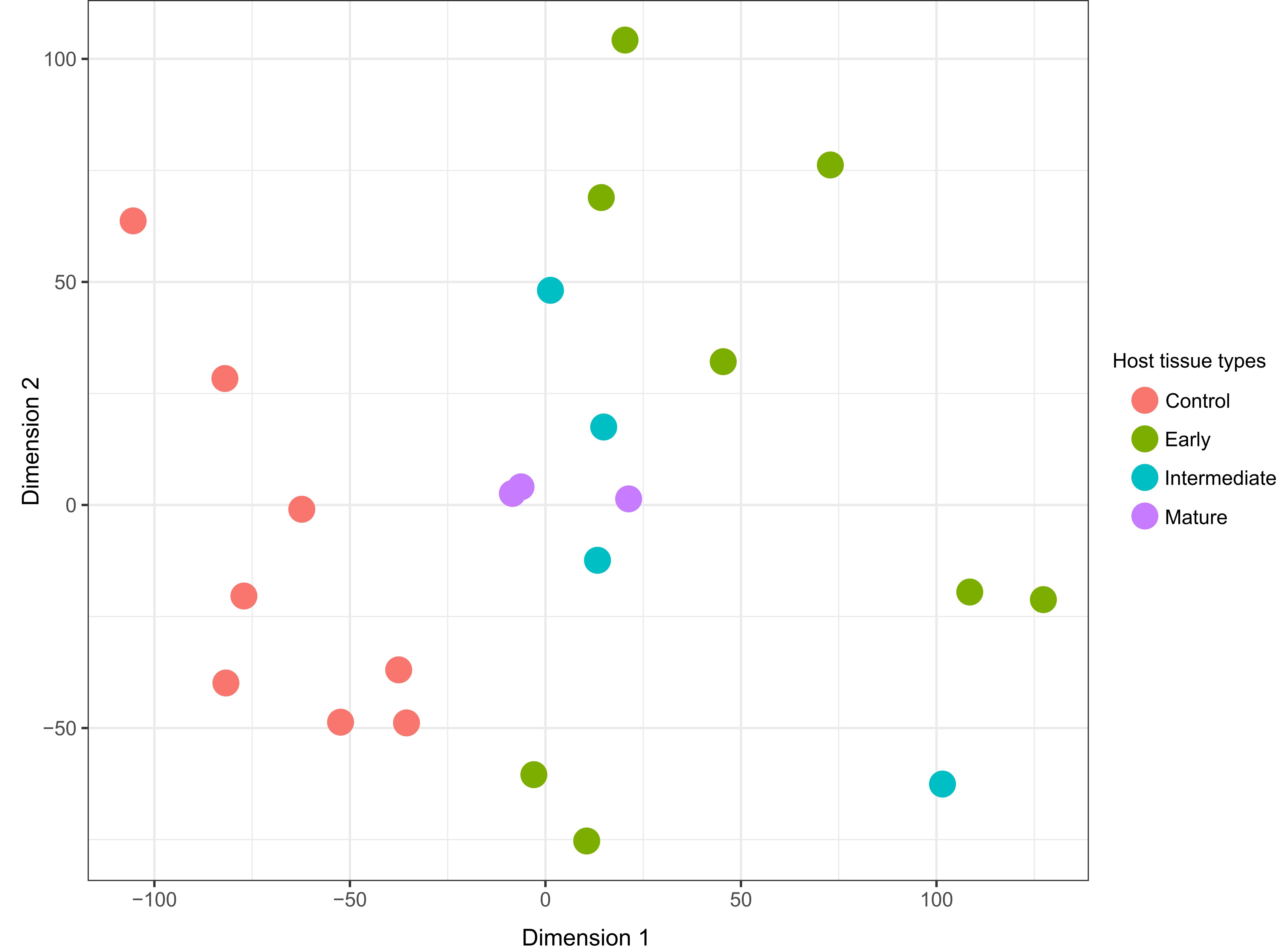

Supplement: Supplementary Figure 5 — Multidimensional scaling (MDS) plot of LCM RNA-Seq data from the host tomato tissues surrounding C. campestris haustoria. “Control” means the tomato stem cortex tissue samples that are not next to C. campestris haustoria, which serve as negative controls in this experiment. The control has eight libraries. The early stage has eight libraries. The intermediate stage has four libraries. The mature stage has three libraries. [file Image_5.JPEG]

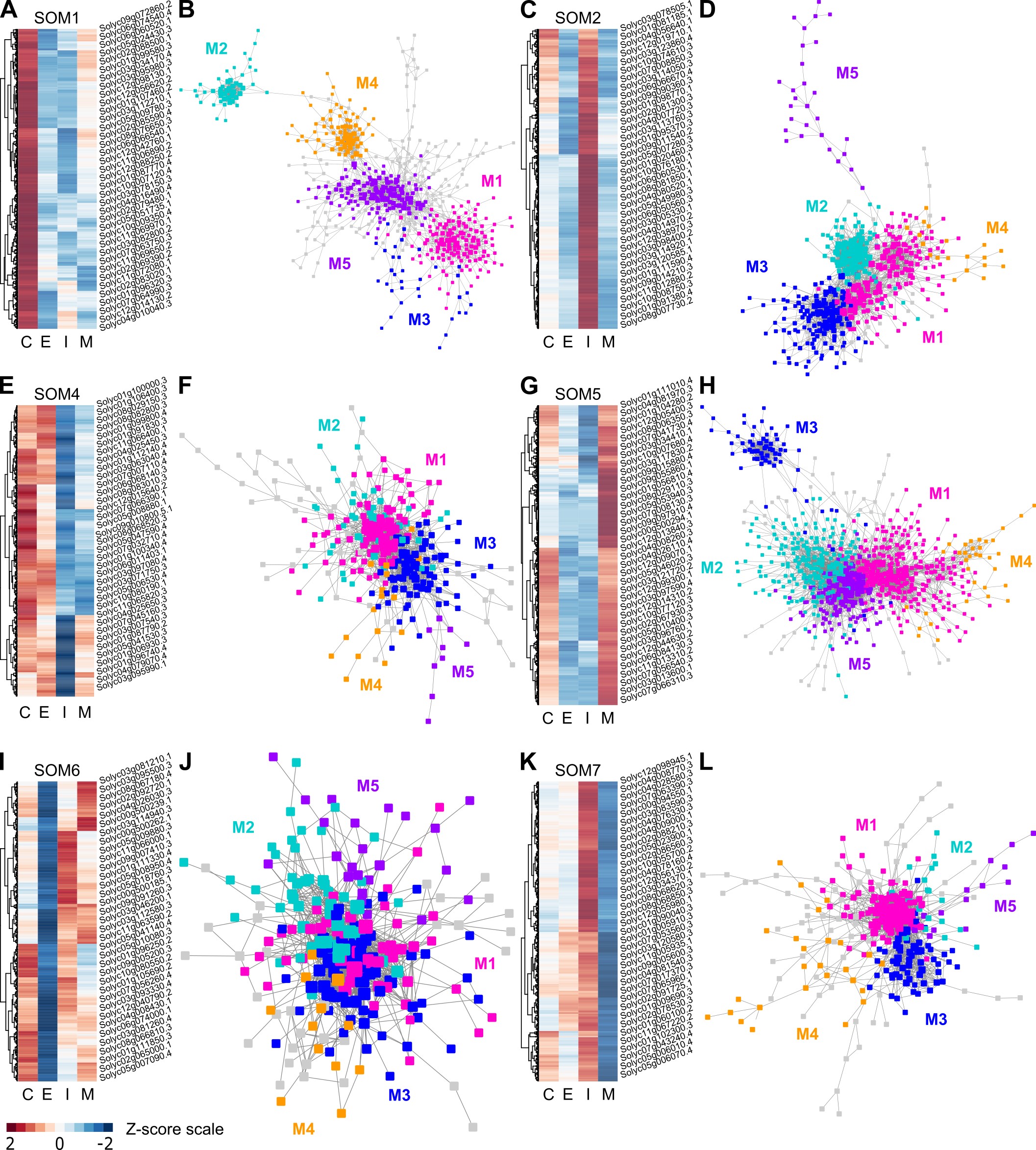

Supplement: Supplementary Figure 6 — Heatmaps and gene-coexpression networks (GCNs) of LCM RNA-Seq data from the host tomato tissues surrounding C. campestris haustoria. (A) A heatmap of gene expression profiles in z-scores for SOM1, which includes genes specifically highly expressed in the control stage. (B) A GCN of genes in SOM1. This SOM1 GCN is composed of five major modules. (C) A heatmap of gene expression profiles in z-scores for SOM2, which includes genes specifically highly expressed in the intermediate stage. (D) A GCN of genes in SOM2. This SOM2 GCN is composed of five major modules. (E) A heatmap of gene expression profiles in z-scores for SOM4, which includes genes specifically highly expressed in the control and early stage. (F) A GCN of genes in SOM4. This SOM4 GCN is composed of five major modules. (G) A heatmap of gene expression profiles in z-scores for SOM5, which includes genes specifically highly expressed in the control and mature stage. (H) A GCN of genes in SOM5. This SOM5 GCN is composed of five major modules. (I) A heatmap of gene expression profiles in z-scores for SOM6, which includes genes specifically repressed expression in the early stage. (J) A GCN of genes in SOM6. This SOM6 GCN is composed of five major modules. (K) A heatmap of gene expression profiles in z-scores for SOM7, which includes genes specifically repressed expression in the mature stage. (L) A GCN of genes in SOM7. This SOM7 GCN is composed of five major modules. The complete gene lists for all SOM units with SOM distances and PCA principal component values are included in Supplementary Table 6. The selected SOM gene lists were used for constructing the GCN based on the expression profiles in tomato LCM data with the following normal quantile cutoffs. The SOM1 GCN cutoff = 0.95. The SOM2 GCN cutoff = 0.93. The SOM4 GCN cutoff = 0.93. The SOM5 GCN cutoff = 0.96. The SOM6 GCN cutoff = 0.94. The SOM7 GCN cutoff = 0.92. [file Image_6.JPEG]

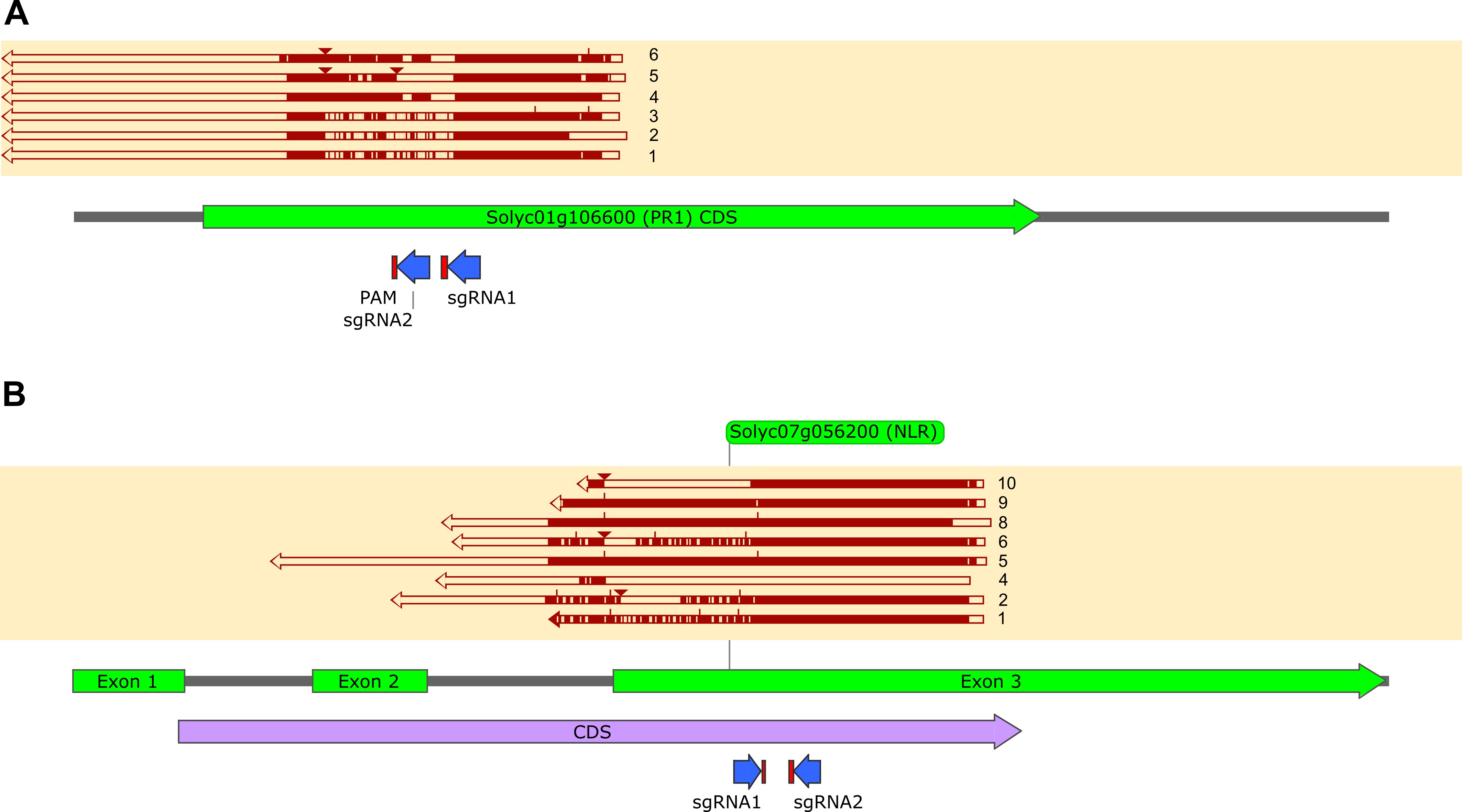

Supplement: Supplementary Figure 7 — Genotyping results of the T1 CRISPR transgenic plants that are used in this study. (A) Genomic DNA PCR product sequencing result of PR1 T1 CRISPR plants. T1 plant #1-3 have similar mixed peaks were present next to the sgRNA1, indicating a biallelic or heterozygous mutation. (B) Genomic DNA PCR product sequencing result of NLR T1 CRISPR plants. T1 plant #1, 2, 6 have similar mixed peaks were present next to the sgRNA1, indicating a biallelic or heterozygous mutation. T1 plant #4 might have a large section of deletion (> 150 bp) next to the two gRNAs, so it has a large section of mismatch and mixed peaks. (A,B) The dark red line indicates the sequencing result. The number next to each dark red line indicates the T1 plant ID number (T1 plant #). The section filled with dark red color indicates regions of perfect sequence match; the empty boxes indicate regions of sequence mismatch. The dark red arrowhead above each sequence indicates a section of insertion. The dark red line above each sequence indicates a single base pair insertion. The gRNAs are labeled as blue arrows. The gRNA sequences that were used in these CRISPR constructs are listed in Supplementary Table 11. The protospacer adjacent motif (PAM) sites are labeled as small red boxes that are right next to gRNAs. Detailed genotyping results of the T1 CRISPR transgenic plants are listed in Supplementary Table 12. [file Image_7.JPEG]
